# muLTi-Arm Therapeutic study in pre-ICu patients admitted with Covid-19-Experimental drugs and mechanisms (TACTIC-E): A structured summary of a study protocol for a randomized controlled trial

**DOI:** 10.1186/s13063-020-04618-2

**Published:** 2020-07-31

**Authors:** Ing Ni Lu, Spoorthy Kulkarni, Marie Fisk, Michalis Kostapanos, Edward Banham-Hall, Sonakshi Kadyan, Simon Bond, Sam Norton, Andrew Cope, James Galloway, Frances Hall, David Jayne, Ian B. Wilkinson, Joseph Cheriyan

**Affiliations:** Clinical Pharmacology, Cambridge University Hospitals NHS Foundation Trust, Cambridge, UK

**Keywords:** COVID-19, Randomised controlled trial, Protocol, Open-label, Adaptive trial, EDP1815, Dapagliflozin, Ambrisentan, Experimental drugs

## Abstract

**Objectives:**

To determine if a specific intervention reduces the composite of progression of patients with COVID-19-related disease to organ failure or death as measured by time to incidence of any one of the following: death, invasive mechanical ventilation, ECMO, cardiovascular organ support (inotropes or balloon pump), or renal failure (estimated Cockcroft Gault creatinine clearance <15ml/min).

**Trial design:**

Randomised, parallel arm, open-label, adaptive platform Phase 2/3 trial of potential disease modifying therapies in patients with late stage 1/stage 2 COVID-19-related disease, with a diagnosis based either on a positive assay or high suspicion of COVID-19 infection by clinical, laboratory and radiological assessment.

**Participants:**

Patients aged 18 and over, with a clinical picture strongly suggestive of COVID-19-related disease (with/without a positive COVID-19 test) AND a risk count (as defined below) >3 OR ≥3 if risk count includes “Radiographic severity score >3”. A risk count is calculated by the following features on admission (1 point for each): radiographic severity score >3, male gender, non-white ethnicity, diabetes, hypertension, neutrophils >8.0 x10^9^/L, age >40 years and CRP >40 mg/L.

Patients should be considered an appropriate subject for intervention with immunomodulatory or other disease modifying agents in the opinion of the investigator and are able to swallow capsules or tablets. The complete inclusion and exclusion criteria as detailed in the Additional file 1 should be fulfilled. Drug specific inclusion and exclusion criteria will also be applied to the active arms. Patients will be enrolled prior to the need for invasive mechanical ventilation, cardiac or renal support. Participants will be recruited across multiple centres in the UK including initially at Cambridge University Hospitals NHS Foundation Trust and St George’s University NHS Foundation Trust. Other centres will be approached internationally in view of the evolving pandemic.

**Intervention and comparator:**

There is increasing evidence of the role of immunomodulation in altering the course of COVID-19. Additionally, various groups have demonstrated the presence of pulmonary shunting in patients with COVID-19 as well as other cardiovascular complications. TACTIC-E will assess the efficacy of the novel immunomodulatory agent EDP1815 versus the approved cardio-pulmonary drugs, Dapagliflozin in combination with Ambrisentan versus the prevailing standard of care.

EDP1815 will be given as 2 capsules twice daily (1.6 x 10^11^ cells) for up to 7 days with the option to extend up to 14 days at the discretion of the principal investigator or their delegate, if the patient is felt to be clinically responding to treatment, is tolerating treatment, and is judged to be likely to benefit from a longer treatment course. Ambrisentan 5mg and Dapagliflozin 10mg will be given in combination once daily orally for up to maximum of 14 days. Patients will be randomised in a 1:1:1 ratio across treatments. Each active arm will be compared with standard of care alone. Additional arms may be added as the trial progresses. No comparisons will be made between active arms in this platform trial.

**Main outcomes:**

The primary outcome is the incidence (from baseline up to Day 14) to the occurrence of the any one of the following events: death, invasive mechanical ventilation, extra corporeal membrane oxygenation, cardiovascular organ support (inotropes or balloon pump), or renal failure (estimated Cockcroft Gault creatinine clearance <15ml/min).

**Randomisation:**

Eligible patients will be randomised using a central web-based randomisation service (Sealed Envelope) in a 1:1:1 ratio, stratified by site to one of the treatment arms or standard of care.

**Blinding (masking):**

This is an open-label trial. Data analysis will not be blinded.

**Numbers to be randomised (sample size):**

There is no fixed sample size for this study. There will be an early biomarker-based futility analysis performed at a point during the study. If this biomarker futility analysis is not conclusive, then a second futility analysis based on clinical endpoints will be performed after approximately 125 patients have been recruited per arm. Provisionally, further analyses of clinical endpoints will be performed after 229 patients per active arm and later 469 patients per arm have been recruited. Further additional analyses may be triggered by the independent data monitoring committee.

**Trial Status:**

TACTIC-E Protocol version number 1.0 date May 27^th^, 2020. Recruitment starts on the 3^rd^ of July 2020. The end trial date will be 18 months after the last patient’s last visit and cannot be accurately predicted at this time.

**Trial registration:**

Registered on EU Clinical Trials Register EudraCT Number: 2020-002229-27 registered: 9 June 2020.

The trial was also registered on ClinicalTrials.gov (NCT04393246) on 19 May 2020.

**Full protocol:**

The full protocol is attached as an additional file, accessible from the Trials website (Additional file 1). In the interest in expediting dissemination of this material, the familiar formatting has been eliminated; this Letter serves as a summary of the key elements of the full protocol.

## Supplementary information

**Additional file 1.** Full Study Protocol.

## Data Availability

Not applicable. Ownership of the data arising from this trial resides with the trial team and the sponsor.

