# Supplementary material for: muLTi-Arm Therapeutic study in pre-ICu patients admitted with Covid-19-Experimental drugs and mechanisms (TACTIC-E): A structured summary of a study protocol for a randomized controlled trial
Source: Trials. 2020 Jul 31;21:690. doi: 10.1186/s13063-020-04618-2 (PMC7393245; doi:10.1186/s13063-020-04618-2)

Clinical Trial Protocol

Trial Title: **mulTi-Arm Therapeutic study in pre-ICu patients admitted with Covid-19 – Experimental drugs and mechanisms (TACTIC-E)**

Protocol Number: TACTIC-E

EudraCT Number: 2020-002229-27

ClinicalTrials.gov Number: NCT04393246

Investigational Products: EDP1815; Ambrisentan and Dapagliflozin

Protocol Version: v1.0 dated 27 May 2020

Chief Investigator: Dr Joseph Cheriyan
CI Address: Cambridge University Hospitals NHS Foundation Trust,
Box 98, Level 3,
ACCI Building,
Cambridge University Hospitals NHS Foundation Trust
Hills Road,
Cambridge,

CB2 0QQ

Telephone: 01223 274915

Trial Sponsor: Cambridge University Hospitals NHS Foundation Trust

SAE Reporting: TACTIC Clinical Trial Coordinator

Email: Cambs.cardiovascular@nhs.net , cctupharmacovigilance@addenbrookes.nhs.uk for all SAE reporting.

1 Protocol Signatures:

I give my approval for the attached protocol entitled "mulTiArm Therapeutic study in pre-ICu patients Admitted with Covid-19 – Experimental drugs and mechanisms (TACTIC-E)" dated 27 May 2020

Chief Investigator

Name: Dr Joseph Cheriyan

Signature:

Date: 27 May 2020

Site Signatures

I have read the attached protocol entitled "mulTiArm Therapeutic study in pre-ICu patients Admitted with Covid-19 – Experimental drugs and mechanisms (TACTIC-E)" dated 27 May 2020 and agree to abide by all provisions set forth therein.

I agree to comply with the conditions and principles of Good Clinical Practice as outlined in the European Clinical Trials Directives 2001/20/EC and 2005/28/EC, the Medicines for Human Use (Clinical Trials) Regulations 2004 (SI 2004/1031) and any subsequent amendments of the clinical trial regulations, the Sponsor's SOPs, and other regulatory requirements as amended.

I agree to ensure that the confidential information contained in this document will not be used for any other purpose other than the evaluation or conduct of the clinical investigation without the prior written consent of the Sponsor

Principal Investigator

Name:

Signature: ____

Date: ____________________________

2 Trial Management Committee(s) and Protocol Contributors

**Chief Investigator:**	
Dr Joseph Cheriyan (Chief Investigator)	Consultant in Clinical Pharmacology, Directorate of Acute Medicine Services, Division C, Cambridge University Hospitals NHS Foundation Trust and Division of Experimental Medicine & Immunotherapeutics, Dept of Medicine, Univ. of Cambridge, Box 98, Hills Road, Cambridge, CB2 0QQ<br><br>Telephone: 01223 336517
Dr James Galloway (Co-Lead)	Senior Lecturer and Honorary Consultant Rheumatologist<br>Centre for Rheumatic Diseases<br>10 Cutcombe Road,<br>King's College London,<br>London. SE5 9RJ
**Sub-Investigators / Protocol Contributors**:	
Dr Frances Hall	Consultant Rheumatologist, Cambridge University Hospitals NHS Foundation Trust Rheumatology Research Unit, Unit E6, Box 194, Hills Road, Cambridge CB2 0QQ
Professor Ian Wilkinson	Head of Division, Division of Experimental Medicine & Immunotherapeutics, University of Cambridge & Director, Cambridge Clinical Trials Unit, Cambridge University Hospitals NHS Foundation Trust, Box 98, Hills Road, Cambridge, CB2 0QQ
Professor Andrew Cope	Versus Arthritis Professor of Rheumatology<br>Head, Centre for Rheumatic Diseases<br>Faculty of Life Sciences and Medicine<br>Guy's Campus<br>King's College London<br>London SE1 1UL
Professor David Jayne	Director of the Vasculitis and Lupus Service and Professor of Clinical Autoimmunity, Division of Experimental Medicine and Immunotherapeutics, University of Cambridge, Hills Road, Cambridge, CB2 0QQ
Professor Iain McInnes	Muirhead Chair of Medicine, Versus Arthritis Professor of Rheumatology, and Director of Institute of Infection, Immunity and Inflammation<br>University of Glasgow<br>Glasgow G12 8TA

Dr Michalis Kostapanos	Cambridge University Hospitals NHS Foundation Trust and Division of Experimental Medicine & Immunotherapeutics, Dept. of Medicine, Univ. of Cambridge, Box 98, Hills Road,<br>Cambridge, CB2 0QQ
Dr Edward Banham-Hall	Clinical Trials Physician, Consultant in Acute Medicine, Directorate of Acute Medical Services, Division C, Cambridge University Hospitals NHS Foundation Trust
**Trial coordination:**	
Ms Sonakshi Kadyan	Cambridge Clinical Trials Unit, Box 401, Cambridge University Hospitals NHS Foundation Trust,<br>Cambridge CB2 0QQ<br>01223 349007<br>sonakshi.kadyan@addenbrookes.nhs.uk

**Trial statisticians:**	
Dr Simon Bond	Cambridge Clinical Trials Unit, Box 401, Cambridge University Hospitals NHS Foundation Trust, Hills Road, Cambridge, CB2 0QQ
Dr Sam Norton	Senior Lecturer in Research Methods & Statistics<br>Centre for Rheumatic Diseases<br>10 Cutcombe Road,<br>King's College London,<br>London. SE5 9RJ
**Trial pharmacist(s):**	
Mrs Anita Chhabra	Central Pharmacy Level 1, Box 55<br>Cambridge University Hospitals NHS Foundation Trust, Hills Road, Cambridge, CB2 0QQ
**Sponsor:**	
Cambridge University Hospitals NHS Foundation Trust	Research & Development Department (Box 277)<br>Addenbrooke's Hospital, Hills Road<br>Cambridge, CB2 0QQ<br>Telephone: 01223 245151 e-mail:<br>research@addenbrookes.nhs.uk

Table of Contents

1 Protocol Signatures: 2
2 Trial Management Committee(s) and Protocol Contributors 3
Table of Contents 5
3 Abbreviations 8
Alkaline phosphatase 8
Alanine transaminase 8
Acute respiratory distress syndrome 8
Aspartate transaminase 8
Gamma glutamyl transpeptidase 8
Granulocyte-macrophage colony-stimulating factor 8
4 Trial Flow Chart 10
5 Introduction 11
5.1 Background 11
5.2 TACTIC-E clinical trial 13
5.3 Selecting strategies for a therapeutic trial in COVID19 13
5.4 Choice of individual drugs 15
5.4.1 Selection of immune modulating agents 15
5.4.2 Selection of agents for end-organ protection and modulation of the pulmonary vascular response – combination SGLT-2 inhibitor and endothelin receptor antagonism 15
5.5 Patient selection for the study 17
5.6 Study proposal 20
5.7 Trial Hypotheses 22
6 Information on Selected Treatments 22
6.1 EDP 1815 22
6.1.1 Mechanism of Action 22
6.1.2 Rationale for use as Therapeutic in COVID-19-related disease 23
6.2 Dapagliflozin 24
6.2.1 Mechanism of Action 24
6.2.2 Rationale for use as Therapeutic in COVID-19-related disease 24
6.3 Ambrisentan 24
6.3.1 Mechanism of Action 24
6.3.2 Rationale for use as Therapeutic in COVID-19-related disease 24
7 Trial Design 26
7.1 Statement of Design 26
7.1.1 Addition of treatment arms 26
7.1.2 Termination of treatment arms 26
7.1.3 Termination for Safety 27
7.1.4 Termination for Futility 27
7.2 Participants Trial Duration 27
7.3 Trial Objectives 28
7.3.1 Primary objective 28
7.3.2 Secondary objectives 28
Figure 8. 7-point Ordinal Scale 28
7.3.3 Exploratory objectives 28
7.4 Trial Outcome Measures 28
7.4.1 Primary outcome measure 28
7.4.2 Secondary outcome measures 29
7.4.3 Exploratory outcome measures 29

8 Selection and withdrawal of participants ... 29
8.1 Inclusion Criteria ... 29
8.2 Exclusion Criteria ... 29
8.2.1 General exclusion criteria ... 29
8.2.2 Drug specific exclusion criteria ... 30
8.2.3 EDP1815 Specific Exclusions ... 30
8.2.4 Dapagliflozin and Ambrisentan Specific Exclusions ... 30
8.3 Treatment Assignment and Randomisation Number ... 30
8.4 Treatment Cessation Criteria ... 31
8.5 Consent withdrawal ... 31
9 Trial Treatments ... 32
Treatment Summary ... 32
9.1 EDP 1815 ... 32
9.2 Dapagliflozin ... 34
9.2.1 Legal status ... 34
9.2.2 Supply ... 34
9.2.3 Packing and Labelling ... 34
9.2.4 Storage conditions ... 34
9.2.5 Maximum duration of treatment of a participant ... 34
9.2.6 Dose ... 34
9.2.7 Administration ... 34
9.2.8 Dose modifications ... 35
9.2.9 Side effects ... 35
9.3 Ambrisentan ... 35
9.3.1 Description ... 35
9.3.2 Legal status ... 35
9.3.3 Supply ... 35
9.3.4 Labelling ... 36
9.3.5 Storage conditions ... 36
9.3.6 Maximum duration of treatment of a participant ... 36
9.3.7 Dose ... 36
9.3.8 Dose modifications ... 36
9.3.9 Side effects ... 36
9.3.10 Additional information ... 37
9.4 Active comparator products ... 37
9.4.1 Missed or replacement doses ... 37
9.5 Accountability and dispensing ... 37
9.5.1 Pharmacy Responsibilities: ... 38
9.5.2 Dispensing ... 38
9.5.3 Drug accountability ... 38
9.5.4 Returns and destruction and treatment compliance ... 38
10 Procedures and assessments ... 38
10.1 Participant identification ... 38
10.2 Consent ... 39
10.3 Screening evaluation ... 39
10.3.1 Screening Assessments (Day -2 to Day -1) ... 39
10.3.2 Participant Randomisation ... 40
10.4 Baseline Assessments (Day -2 to Day -1) ... 40
10.5 Trial assessments ... 41
10.5.1 Timing of in hospital assessments ... 41
10.5.2 Further assessments and end of trial visit ... 41
10.6 Schedule of Assessments ... 44
10.7 End of Trial Participation ... 47
10.8 Trial restrictions ... 47

11 Assessment of Safety 48
11.1 Definitions 48
11.1.1 Adverse event (AE) 48
11.1.2 Adverse reaction to an investigational medicinal product (AR) 48
11.1.3 Unexpected adverse reaction 48
11.1.4 Serious adverse event or serious adverse reaction (SAE / SAR) 48
11.1.5 Suspected Unexpected Serious Adverse Reaction (SUSAR) 49
11.1.6 Reference Safety Information (RSI) 49
11.2 Expected Adverse Reactions/Serious Adverse Reactions (AR /SARs) 49
11.3 Evaluation of adverse events 49
11.3.1 Assessment of seriousness 49
11.3.2 Assessment of causality 49
11.3.3 Clinical assessment of severity 50
11.3.4 Recording of adverse events 50
11.4 Recording and Reporting SAEs and SARs 50
11.5 Reporting of Suspected Unexpected Serious Adverse Reactions (SUSARs) 51
11.5.1 Who should report and whom to report to? 51
11.5.2 When to report? 51
11.5.3 Fatal or life-threatening SUSARs 51
11.5.4 Non-fatal and non-life-threatening SUSARs 52
11.5.5 Method and timelines of SUSAR reporting 52
11.5.6 Minimum criteria for initial expedited reporting of SUSARs 52
11.5.7 Follow-up reports of SUSARs 52
11.5.8 Format of the SUSARs reports 52
11.6 Pregnancy Reporting 52
11.7 Adverse events of special interest (AESIs) 53
12 Toxicity – Emergency Procedures 53
13 Evaluation of Results (Definitions and response/evaluation of outcome measures) 53
13.1 Response criteria 53
14 Storage and Analysis of Samples 53
15 Statistics 53
15.1 Statistical methods 53
15.2 Interim analyses 54
15.2.1 Ongoing Safety Monitoring 54
15.2.2 Early Biomarker Analysis 54
15.2.3 Clinical Efficacy Interim Analyses 54
15.3 Number of Participants to be enrolled 55
15.4 Procedure to account for missing or spurious data 56
15.5 Definition of the end of the trial 56
16 Data handling and record keeping 57
16.1 CRF 57
16.2 Source Data 57
16.3 Data Protection & Participant Confidentiality 57
17 Independent Data Monitoring Committee 58
18 Trial Steering Committee 58
19 Ethical & Regulatory considerations 58
19.1 Ethical committee review 58
19.2 Regulatory Compliance 59
19.3 Protocol Amendments 59
19.4 Peer Review 59
19.5 Declaration of Helsinki and Good Clinical Practice 59
19.6 GCP Training 59
20 Sponsorship, Financial and Insurance 59

21 Monitoring, Audit & Inspection 60
22 Protocol Compliance and Breaches of GCP 60
23 Publications policy 60
24 References 61
Safety Reporting Flow Chart 643

3 Abbreviations

Ab	Antibody
ACE	Angiotensin Converting Enzyme
ADCC	Antibody dependent cell-mediated cytotoxicity
AE/AR	Adverse event/Adverse Reaction
AESI	Adverse event of special interest
ALP	Alkaline phosphatase
ALT	Alanine transaminase
ARDS	Acute respiratory distress syndrome
AST	Aspartate transaminase
BD	Twice daily
CA	Competent Authority
CAD	Coronary artery disease
CH	Clonal Hematopoiesis
COVID-19	Severe Adult Respiratory Syndrome Coronavirus 2
CRC	COVID-19 related complications
CRF	Case Report Form
CRP	C reactive protein
CTIMP	Clinical Trial of Investigational Medicinal Product
CTL	Cytotoxic T Lymphocyte
CYP	Cytochrome P450
DAD	Diffuse Alveolar Damage
DMARD	Disease-modifying anti-rheumatic drug
DC	Dendritic Cell
IDMC	Independent Data Monitoring Committee
DNA/RNA	Deoxyribonucleic acid / Ribonucleic acid
DSUR	Development Safety Update Report
DVT	Deep venous thrombosis
ECMO	Extracorporeal membrane oxygenation
FDA	Food and Drug Administration
G-CSF	Granulocyte-colony stimulating factor
GP	General Practitioner
GCP	Good Clinical Practice
GGT	Gamma glutamyl transpeptidase
GM-CSF	Granulocyte-macrophage colony-stimulating factor
HBsAg	Hepatitis B surface antigen
HCV	Hepatitis C virus
HLH	Haemophagocytic Lymphohistiocytosis
IB	Investigator Brochure
ICF	Informed Consent Form

ICU	Intensive Care Unit
IFN	Interferon
IL	Interleukin
IMP	Investigational Medicinal Product
IUD	Intrauterine Device
JAK	Janus Kinase
LDH	Lactate Dehydrogenase
LFT	Liver/lung function test
LPS	Lipopolysaccharide
MASP	Mannan-binding lectin serine protease
MedDRA	Medical Dictionary for Regulatory Activities
MERS	Middle East Respiratory Syndrome
MHRA	Medicines and Healthcare products Regulatory Agency
MRC	Medical Research Council
MTX	Methotrexate
NK	Natural Killer cell
NIMP	Non Investigational Medicinal Product
NLR	Neutrophil:Lymphocyte Ratio
NT-proBNP	N-terminal pro B type natriuretic peptide
PBMC	Peripheral Blood Mononuclear Cells
PC	Plasma Cell
PCR	Polymerase Chain Reaction
PDGF	Platelet Derived Growth Factor
PE	Pulmonary embolism
PIS	Participant Information Sheet
po	Per oral
qtPCR	Quantitative Polymerase Chain Reaction
R&D	Research and Development
RA	Regulatory Agency
REC	Research Ethics Committee
RSI	Reference Safety Information
rtPCR	Real Time Polymerase Chain Reaction
SAE/SAR	Serious Adverse Event/Serious Adverse Reaction
SBECD	Betadex Sulfobutyl Ether Sodium
SC	Subcutaneously
sHLH	Secondary Haemophagocytic Lymphohistiocytosis
SoC	Standard of Care
STAT	Signal Transducer and Activator of Transcription
SUSAR	Suspected Unexpected Serious Adverse Reaction
TB	Tuberculosis
TGF	Tissue Growth Factor
TMG	Trial Management Group
TNF	Tissue Necrosis Factor
TPM	Trial Procedures Manual
TRC	Translational Research Collaboration
TSC	Trial Steering Committee
VTE	Venous thromboembolism
WHO	World Health Organisation

4 Trial Flow Chart

This trial is a randomised, parallel arm, open-label platform trial.

Screening (Day -2 to -1)
Aged 18 or over, Confirmed / Clinically Suspected COVID, Risk score for progression >3*(or ≥3 if radiological score included)

Baseline (Day -2 to -1)
Core data collection, research sampling

Randomisation

- Standard of care**
- EDP1815^ 2 capsules BD + Standard of Care**
- Ambrisentan 5mg and Dapagliflozin^ 10mg OD+ Standard of Care**
- Potential additional arms (after ethics and regulatory approval)

In hospital follow up
Core data collection (daily for 14 days or until death / discharge)***
Research sampling (days 2, 6, 14/discharge***)
Adverse event data (including SAE / SAR / SUSAR)

Day 28 (+/- 7 days) **follow up**
Limited data collection

Day 90 (+/- 7 days) **follow up**
Limited data collection

* Risk score defined in protocol
** Standard of Care may include approved anti-virals
*** Assessments at day 14 or discharge will be done where feasible
^ Each arm is assessed separately against the standard of care arm and may be stopped for futility or safety independent of the other treatment arms

5 Introduction

5.1 Background

The COVID-19 pandemic, as declared on 11th March 2020 by the World Health Organisation (WHO), is caused by a novel coronavirus (SARS-Cov-2). In the UK on 17/4/20 108,692 cases had a laboratory-confirmed diagnosis and the total number of COVID-19 associated deaths in hospital totalled 14,576; these data do not cover cases and deaths in the community, where, as yet, no testing is available.

The majority of individuals infected with COVID19 appear to have mild/moderate symptoms but ~15% have severe disease and there is ~2% mortality across the population. Identified risk factors for severe COVID-19-related disease include age, cardiovascular disease, diabetes and male gender. COVID19-related complications (CRC) include acute respiratory distress syndrome, arrhythmia, shock, acute kidney injury, acute cardiac injury, liver dysfunction and secondary infection [Huang et al 2020; King’s Critical Care Evidence Summary 09/03/2020]. Currently, there are no vaccines, prophylactic agents, nor therapeutic agents of proven efficacy. The international research community is rapidly accumulating information regarding the pathogenesis of COVID19 infection. The following key themes have emerged to date:

- Early infection is often asymptomatic but these individuals can be infectious.
- A significant proportion of the severe symptoms and life-threatening complications of these viral infections are not driven by primary viral infection but by an excessive host immune response to COVID-19.
- The immune response to COVID19 is more often damaging in older adults and in patients with selected comorbidities.
- Severe organ damage in association with COVID19 is accompanied by a dysregulated autoinflammatory and autoimmune syndrome (see Figure 1).

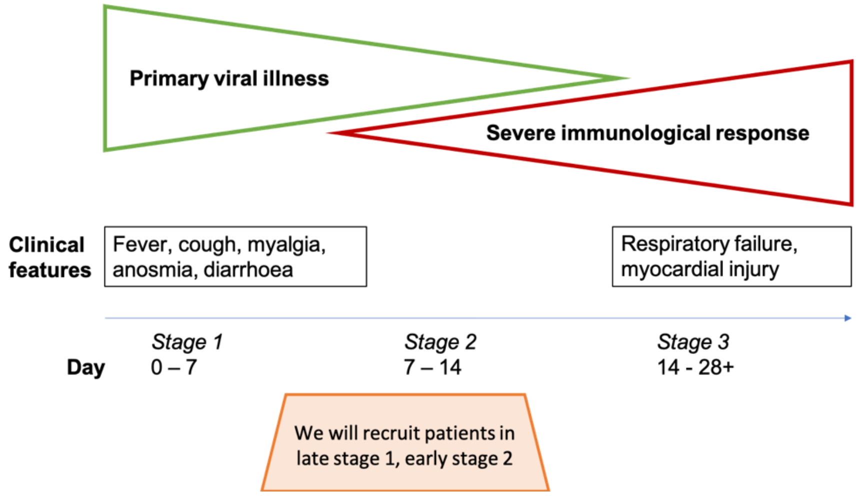

Figure 1: Stages of COVID19

<u>Virology</u>
Coronaviruses are large, enveloped RNA viruses that are distributed widely among mammals and birds. Previous outbreaks of severe respiratory disease caused by coronavirus infections in humans have followed zoonotic transmission. SARS-CoV

was identified in 2003 and MERS-CoV emerged in 2012. COVID-19 or SARS-CoV2 is closely related to SARS-CoV and, both use ACE2 as the receptor for the envelope spike protein of COVID19 and is the means of the virus binding to its target cells. Coronaviruses exhibit a range of strategies for evading the early innate immune response, for example by inhibiting the action of type I interferons. This facilitates early viral replication and reduces systemic symptoms in the host.

Cytokine Dysregulation in COVID-19-related Disease (Figure 2)

Based on the SARS epidemic, whilst SARS-CoV2 infection evades detection by the immune system in the first 24h of infection, it ultimately induces a massive immune system effector response in the subgroup of people who develop severe COVID19-related disease. This leads to potentially life-threatening lung and sometimes multi-organ damage. It is important to note that the development of Diffuse Alveolar Damage (DAD) is often independent of high-titre viral replication (Peiris et al 2003). The massive immune and inflammatory response in affected lungs includes production of high levels of IL-6, TNF-alpha and IL-1-beta, as well as influx of neutrophils and cytotoxic T cells. A Th2 (IL4, IL13) response from alternatively-activated macrophages, and an associated profibrotic phenotype (including increased TGF-beta and PDGF-alpha production) leads to lung fibrosis. Activation of the coagulation cascade is associated with development of fibrin clots in the alveoli. (Gralinski and Baric 2015).

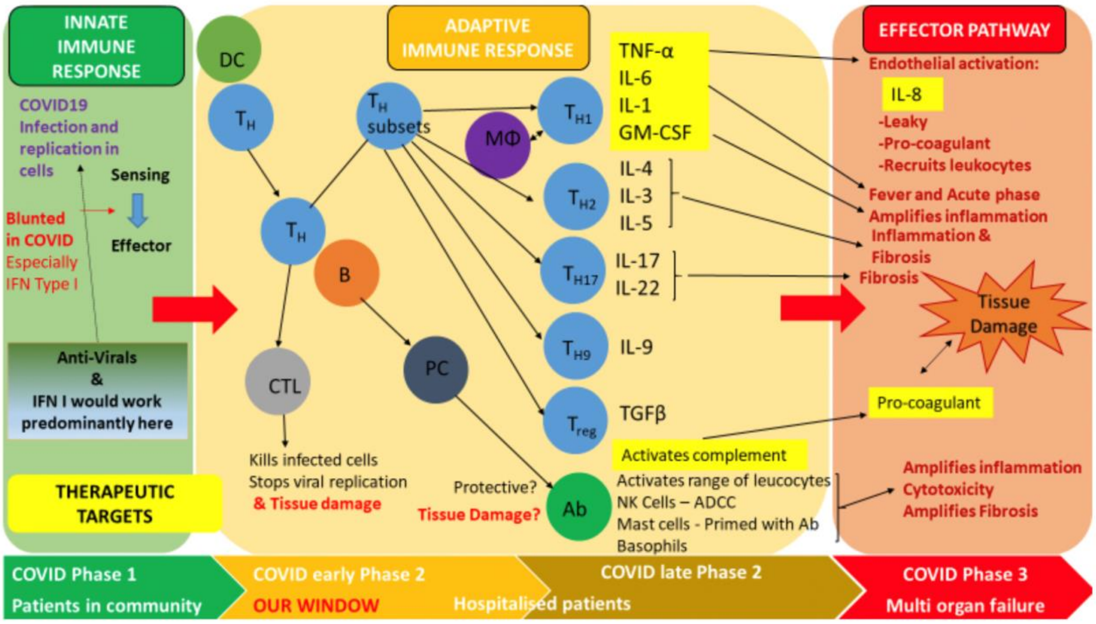

Figure 2: COVID-19 immunopathology and potential therapeutic targets
PC indicates plasma cell, DC-dendritic cell, Ab-antibody, CTL-Cytotoxic T lymphocyte, NK natural killer cells, ADCC-antibody dependent cell-mediated cytotoxicity. TH- T helper cell, IL- interleukin, GM-CSF- Recombinant Human Granulocyte Macrophage Colony-Stimulating Factor

Pulmonary shunting in COVID-19 leading to hypoxia

Gattinoni et al describe the phenomenon of early COVID-19 pneumonia associated with prominent pulmonary shunting. This has been described as the “Type L” clinical picture.

This is characterised by the following characteristics:

• Low elastance: the nearly normal compliance indicates that the amount of gas in the lung is nearly normal.
• Low ventilation to perfusion (VA/Q) ratio: since the gas volume is nearly normal, hypoxemia may be best explained by the loss of regulation of perfusion and by loss of hypoxic vasoconstriction. Accordingly, at this stage, the pulmonary artery pressure, should be near normal.
• Low lung weight: Only ground-glass densities are present on CT scan, primarily located subpleurally and along the lung fissures. Consequently, lung weight is only moderately increased.
• Low lung recruitability: the amount of non-aerated tissue is very low, consequently the recruitability is low

Pulmonary shunting and hypoxaemia may offer an additional opportunity for intervention given the prominent role in shunting played by elevated endothelin-1, although fluid retention associated with endothelin antagonists and hepatotoxicity with some agents have limited their use in the treatment of ARDS.

5.2 TACTIC-E clinical trial

This study will assess the efficacy of novel therapeutic agents or novel combinations of approved agents that target dysregulated host response mechanisms that drive the severe lung, and other organ, damage. We will compare each therapy independently to standard-of-care.

By collecting samples for pharmacological and disease biomarkers, the study aims to initially assess the effect of the active therapy on their associated pharmacological biomarkers (if available), and COVID-19 disease biomarkers, to enable early futility analyses to be performed. As there are limited data on the expected variability on COVID19 disease biomarkers, an initial feasibility analysis will be conducted on samples from the study prior to finalising the parameters of the futility analysis. If the feasibility analysis suggests a futility analysis is not possible with fewer than 100 subjects dosed per arm, then the clinical endpoints will be used to perform futility analyses after 125 and 229 subjects per arm have been recruited and dosed.

The medications investigated for efficacy currently in this protocol are EDP1815, and a combination of dapagliflozin and ambrisentan. The anticipated sample size is up to 469 patients per active arm (assuming futility analyses are passed), randomised in a 1:1:1 ratio across treatments.

5.3 Selecting strategies for a therapeutic trial in COVID19

TACTIC-E is an adaptive Phase 2/3 platform clinical trial which aims to test:
(a) unlicensed compounds,
(b) novel combination strategies of licensed drugs
(c) novel treatment approaches with licensed drugs;

to provide:
(a) an initial evaluation of drug safety and potential for clinical benefit using disease and drug-specific biomarkers, and then
(b) determination of clinical efficacy in drugs which demonstrate sufficient potential benefit and safety.

Drugs will initially be evaluated against safety and potential efficacy using clinical disease severity biomarkers, and, where applicable drug specific biomarkers, at an early stage. Only drugs which appear safe and show potential efficacy will continue for determination of clinical efficacy.

TACTIC-E is therefore focused on the following paradigms:

1. Unlicensed drugs currently in clinical development with sufficient pre-clinical and clinical data to suggest a positive benefit-risk ratio in the treatment of COVID-19
2. Novel combinations of therapies; or Novel approaches to treating COVID19 complications
3. Careful ongoing safety analysis by the IDMC
4. Early futility analysis using biomarkers of pharmacodynamic effect and benefit as well as more standard clinical measures
5. Prompt immune modulatory therapy (e.g. cytokine suppression).
6. Adjuvant therapy to protect against end-organ damage (e.g. modulation of abnormal pulmonary vascular responses to COVID-19 infection).
7. Patient stratification for individuals with early (stage 1 / 2) disease but at high risk of complications.

5.4 Choice of individual drugs

5.4.1 Selection of immune modulating agents

There are important clues from the COVID-19 clinical syndrome that influence decisions. First, it is notable that severe immunological diseases are less common in the elderly. In contrast, the severe phase of COVID-19 preferentially affects older persons. This supplies important clues to the immunological drivers and understanding of the changes associated with immune senescence are helpful. Secondly, the severe phase of COVID-19 develops predictably around 8-14 days into the illness, a time point at the interface between innate and adaptive immunological responses, and when the viral replication may be on the decline.

Third, published cytokine data through the course of disease show a host inflammatory response that has similarities (e.g. extreme ferritin elevation, IL-6 production, endovascular damage and microvascular thrombotic disease) to known diseases such as haemophagocytic lymphohistiocytosis (HLH) and haemolytic uraemic syndrome (HUS), a disease known to complicate other infections. Furthermore, predictors of fatality from a retrospective, multicentre study of 150 COVID-19 cases from Wuhan, China included typical HLH markers: raised D-dimer, ferritin, LDH, troponin, IL-6 and increased NLR ratio (Zhou et al 2020). This is consistent with organ damage caused by a coronavirus-driven excessive autoinflammatory response.

Data from the MERS coronavirus outbreak in 2012 provides some evidence for efficacy of using immune suppressants, with reports of therapeutic benefit from several immunomodulatory medications (Al-Omari et al 2018; Gautret et al 2020). However, corticosteroids were unhelpful and clinical evidence does not support corticosteroid treatment for 2019-nCoV lung injury (Russell et al 2020).

Several studies have already launched using cytokine blockade (including a large European anti-IL-6 trial). However, it is important to acknowledge that it is unclear which specific cytokine is responsible for the inflammatory process in COVID-19. It is therefore desirable to consider a strategy that offers blockade of more than one cytokine pathway. EDP 1815 offers this potential, reducing excessive excursions of multiple key cytokines including IL-6, and hence we have carefully selected this agent as an immune system modulator. This is discussed further in section 6.1.

5.4.2 Selection of agents for end-organ protection and modulation of the pulmonary vascular response – combination SGLT-2 inhibitor and endothelin receptor antagonism

Recent information on patients at risk for developing serious complications, including death, in the setting of COVID-19, indicate that those with cardiometabolic disease (obesity, hypertension, T2DM, atherosclerotic cardiovascular disease, HF, and/or kidney disease at baseline) are at much greater risk (Arentz et al 2020, Grasselli et al 2020).

Gattinoni et al also describe the “L” phenotype of COVID-19 related respiratory failure, where there is prominent hypoxia and pulmonary shunting in the absence of marked radiographic changes consistent with ARDS. This may represent an important earlier opportunity to intervene in the progressive respiratory failure which is seen in association with COVID-19 infection. Utilising an endothelin-A (ET-A) receptor antagonist should reduce pulmonary arterial hypertension, pulmonary shunting to hypoxic areas of the lung, and blunt inflammatory activity.

Data from cell culture suggests that interferon-gamma and TNF-alpha, inflammatory markers upregulated in ARDS drive endothelin-1 production(Shinagawa et al. 2017). Endothelin-1 levels are known to be elevated in patients with ARDS, and reduced clearance of endothelin-1 is also recognised. Persistently elevated endothelin-1 is associated with a worse outcome in patients diagnosed with ARDS (Druml et al. 1993). Pre-clinical models of endothelin antagonism in acute lung injury models support this mode of action with respect to intervention in ARDS. Tezosentan, a non-selective endothelin antagonist attenuated lung injury in endotoxaemic sheep and LU-135252, an ET-A selective receptor antagonist improved oxygenation in an experimental pig model of acute lung injury. In a rat airway model of LPS induced lung damage, both mixed and ET-A selective antagonists were shown to reduce microvascular leakage. Interestingly, an endothelin-B (ET-B) selective antagonist showed no effect in that rat model indicating that an ET-A selective antagonist could be beneficial (Kuklin et al. 2005; Boemke et al. 2000; Hele et al. 2000)

Limited data from use of Bosentan (dual ETA/ETB receptor antagonist) in humans supports a positive effect on pulmonary hypertension and refractory hypoxaemia in a patient with ARDS and H7N9 influenza virus infection. The patient treated with Bosentan had a rapid and sustained improvement in right ventricular systolic pressure which was followed by a gradual improvement in oxygenation (Guo et al. 2014).

Ambrisentan has greater selectivity for the ET-A receptor compared to bosentan. This has a significant potential advantage in ARDS, maintaining the positive effects of ET-1 at the ET-B receptor including vasodilatation, reduced pulmonary shunting and anti-inflammatory effects and clearance of ET-1 itself which is mediated by the ET-B receptor.

Ambrisentan is administered in conjunction with dapagliflozin both to offset any negative effects of fluid re-distribution in the COVID-19 population associated with ET-A receptor antagonist therapy, and because patients may derive further benefit from dapagliflozin therapy.

Empagliflozin is structurally similar to dapagliflozin and has been shown in a rat model of pulmonary hypertension to reduce mean pulmonary artery pressure and mortality, (median survival 24 days for vehicle vs 33 days for empagliflozin) (Chowdhury et al. 2019).

Patients presenting with ARDS may have abnormalities of both cardiac and renal function including increased intra-glomerular pressure, hypertension, and fluid/sodium overload. Existing data in patients with chronic cardiac and or renal dysfunction demonstrate that SGLT2 inhibition can improve all these abnormalities (McMurray et al 2019, Neal et al 2017, Perkovic et al 2019, Zelniker et al 2019).

Although concerns are raised about excessive sodium and water loss in some patients treated with SGLT-2 inhibitors, it appears that the level of diuresis seen is proportionate to the level of fluid overload at baseline (Ohara et al 2020).

The combination of ambrisentan and dapagliflozin is therefore expected to have a significant positive impact on patients with COVID-19 infection presenting with significant respiratory dysfunction.

5.5 Patient selection for the study

Evidence from the field of autoimmune diseases shows that most immune modulation strategies are accompanied by small but significant risks of infection. In the context of COVID19, studies will need to recruit patients who are older, with existing comorbidities (as these are risk factors for COVID19) and with active viral infection. Therefore, it is crucial to select patients in whom the risk of progression to stage 3 disease is sufficient to create clinical equipoise about interventions designed to modulate immune function and/or drive organ protection.

One of the key reasons for selecting EDP1815 as a therapeutic arm in this study is due to its safety profile, which would be particularly beneficial in the COVID19 population as there is no evidence that it increases the risk of infection due to its novel and unique mechanism of action.

It must be acknowledged that immune modulation is not without risk, and that dapaglifozin / ambrisentan may lead to changes in fluid handling and have adverse effects on glucose metabolism.

Retrospective data from the COVID19 outbreak in Wuhan, China have revealed divergence of a range of biomarkers, in the early and mid-phases of infection, which discriminate between survivors and non-survivors (Zhou et al 2020); Several of these (D-dimer, neutrophil:lymphocyte Ratio (NLR), ferritin, LDH), diverge early in the clinical course, and IL6 later (Figure 4).

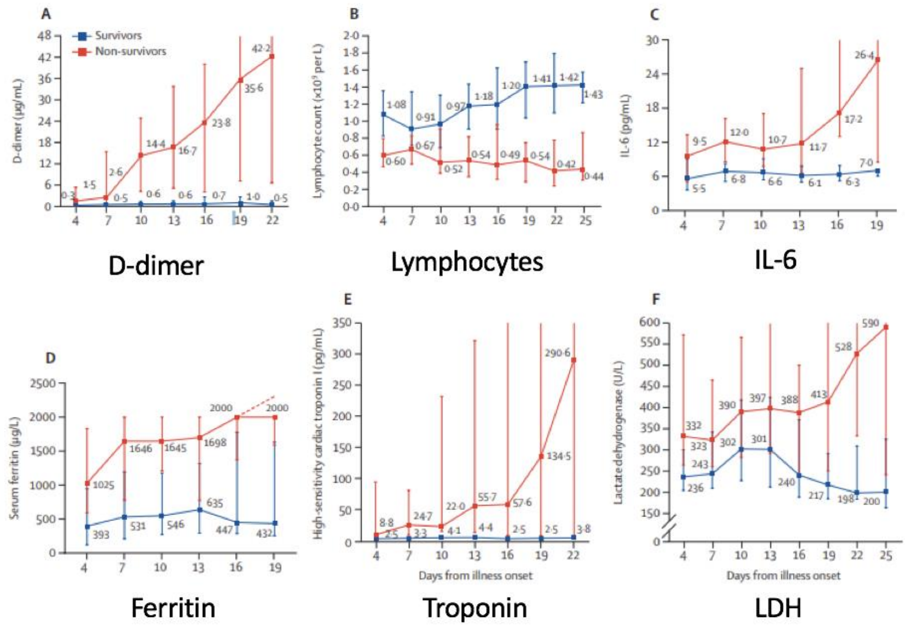

Zhou et al. 2020

Figure 4: Comparison of Biomarkers in Survivors versus Non-survivors from Wuhan Outbreak of COVID-19

Data from the first 200 patients admitted to King's College Hospital (Sneep et al. *under review*) have been used to inform patient selection. Clinical and laboratory data, were modelled using penalised (LASSO) logistic regression to select variables with the most prognostic value. The following variables were considered: Age, gender, non-white ethnicity, radiographic severity on chest radiograph, diabetes, hypertension, neutrophils, lymphocytes, CRP. The radiological severity score was calculated using the method described by Wong et al, 2020. A score of 0-4 was assigned to each lung depending on the extent of involvement by consolidation or ground glass opacities. 0 = no involvement, 1 = <25%, 2 = 25 - 49%, 3= 50 - 75%, 4 = >75% involvement. The scores for each lung were summed to produce a final severity score ranging from 0-8. Radiographs were scored by two emergency department clinicians after a brief training. Interrater reliability was 90.5%.

The outcome modelled was either admission to ICU or death during follow up. The variables selected from the LASSO model were: radiographic severity, male gender, CRP, non-white ethnicity, diabetes, hypertension, and neutrophils (AUC=.86). Age was also selected since it was predictive in a non-linear manner (attributable to a much lower rate of ICU admission with older patients) with an inflection point around 50 years of age. For CRP and Neutrophils the association was linear on the logarithmic scale with no clear threshold effect. For simplicity and pragmatic reasons, cut points were selected for continuous variables at points where risk of poor outcome was consider clinically important. From all the variables, radiographic severity was by far the strongest predictor of progression.

A risk count was calculated by summing (i.e. patients receive 1 point for) each of the following features on admission:

- Radiographic severity score >3

- Male gender
- Non-white ethnicity
- Diabetes
- Hypertension
- Neutrophils >8.0 x10^9/L
- Age >40 years
- CRP >40 mgl/L

The corresponding risks of ICU admission or death in the KCH sample associated with this score are shown in the figure below. All admissions presented with at least one of these risk factors (mean 4.4). 83% scored 3 or above (probability of admission to ICU or death = 13%), 65% scored 4 or above (probability = 21%).

Based upon these data, and the importance of radiographic severity, we selected a score of 3 if radiographic severity score is met, or 4 or higher otherwise as a threshold that captures patients with sufficient risk of progression to justify the risks of immune modulation. This accounted for 71% of the KCH sample. Specifically, individuals meeting this criterion had a 39% risk of admission to ICU or death, versus 9% in those that did not (odds ratio = 5.9).

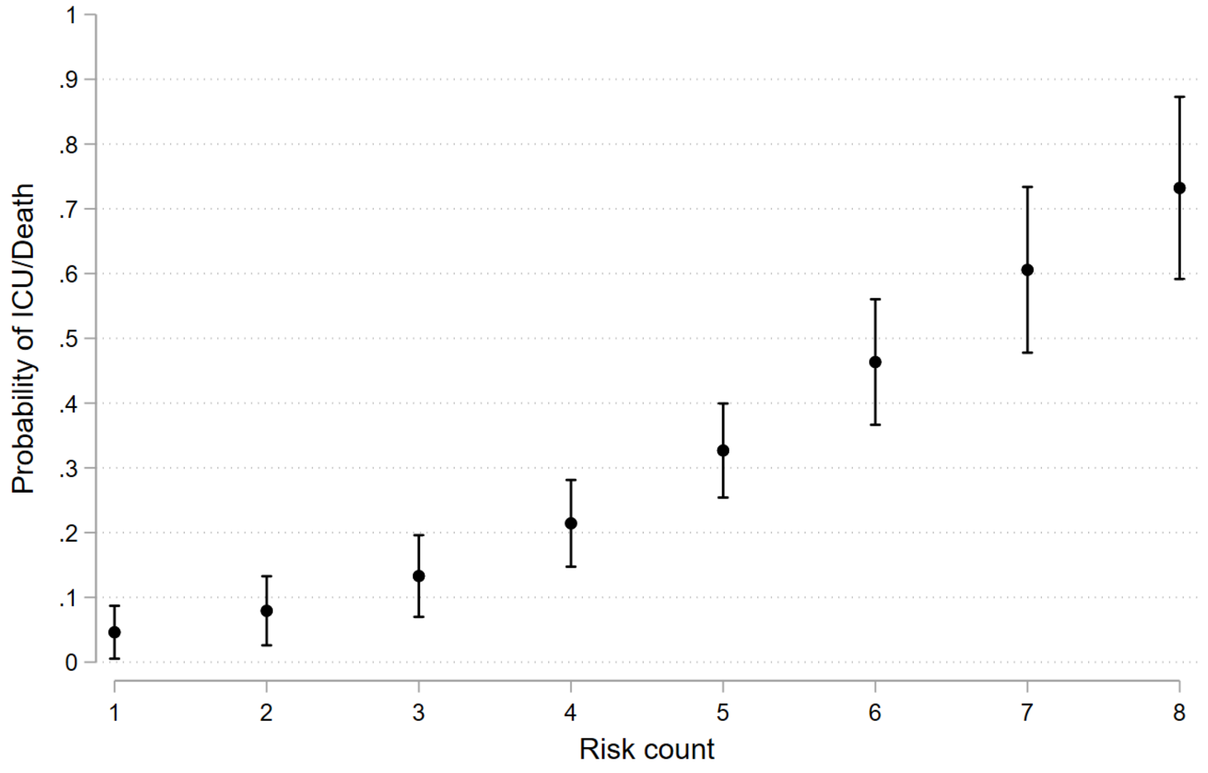

Figure 5: Risk of death or ICU admission. AUC for model = 0.75.

5.6 Study proposal

The study proposal is to assess the safety and efficacy of (1) immunomodulatory agents that target the dysregulated immune response that drives the severe lung, and other organ, damage, and (2) agents which modulate the vascular response to COVID-19. We will compare these to standard-of-care only. TACTIC-E will use a platform design to allow new potential treatments to be investigated through the use of careful ongoing safety monitoring and the use of disease biomarkers to allow early futility analysis. At the same time, the interim analysis enables efficient decisions to be made about safety and futility of treatments, and recruitment can be stopped early if required.

The initial medications investigated for safety and efficacy in this protocol are EDP1815, and the combination of Ambrisentan and Dapagliflozin.

The maximum anticipated sample size is up to 469 patients per arm, randomised in a 1:1 ratio against standard of care.

Safety of patients (AESI, AR, SAE, SAR, SUSAR) will be carefully monitored on an ongoing basis with the CI or delegate reviewing the safety data in each cohort weekly. The IDMC will review the data after the first 10 subjects have reached Day 14/discharge/primary endpoint in each of the arms. This will be repeated after 30 per arm have reached Day 14/discharge/primary endpoint in each of the arms and again at 100 subjects per arm. The timings of subsequent analyses will be determined by the IDMC. In addition, the CI will call an *ad hoc* meeting at any point to review the data from an individual cohort if they are concerned about a potential safety signal.

We will assess potential clinical benefit/futility early in the trial using changes in disease biomarkers. We will estimate the number of subjects required to undertake this analysis in an adequately powered manner using data (change and SD of change) from the first 30 subjects in each arm who have completed 14 days/discharge/met the primary endpoint. These analyses will be detailed in the IDMC charter and overseen by the IDMC. Details of this interim analysis are described in further detail in section 15.2. If it is not feasible to use a biomarker driven approach (due to insufficient power) then clinical endpoints will be used to assess futility after 125 subjects have been enrolled in each arm have completed 14 days/discharge/met the primary endpoint.

TACTIC-E Trial design

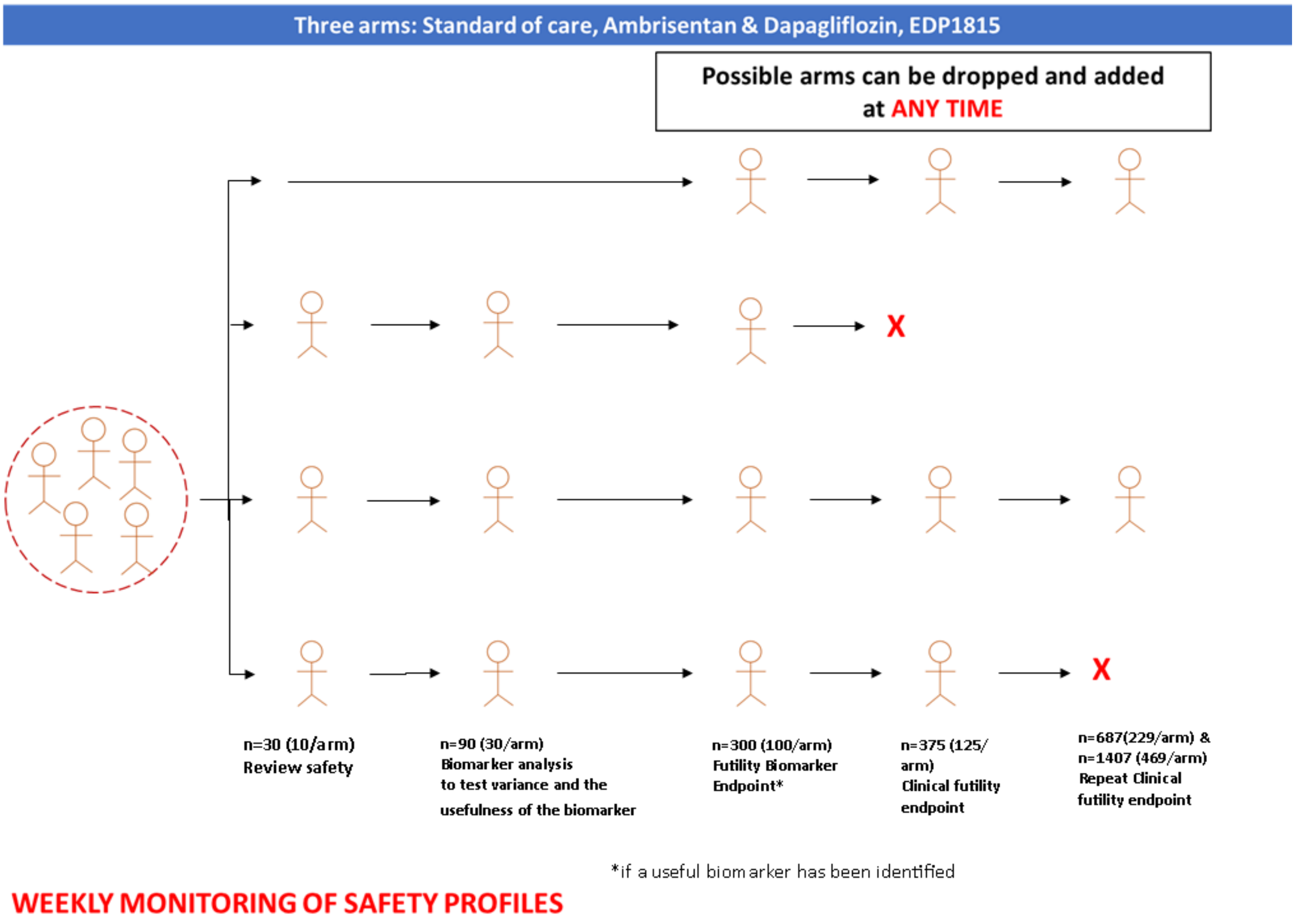

Figure 6: Platform design

5.7 Trial Hypotheses

We aim to test the hypotheses that:

- Active treatment reduces progression to organ failure or death COVID-19 in patients at late stage 1/early stage 2 disease, compared to standard of care

- Clinical and biochemical markers can be used to stratify each patient to an effective therapeutic agent and can report early on efficacy of the therapeutic approach.

6 Information on Selected Treatments

6.1 EDP 1815

6.1.1 Mechanism of Action

EDP1815 is an orally administered pharmaceutical preparation of a single strain of Prevotella histicola isolated from the duodenum of a human donor. Prevotella is the genus of human commensal organisms commonly found on oral, nasopharyngeal, gastrointestinal, and genito-urinary mucosal surfaces. It has been found in all human population groups studied so far. EDP1815 is currently in phase 2 clinical development and has European and US approval to initiate a multinational psoriasis study, scheduled for 2Q2020.

Preclinical and clinical data have demonstrated that EDP1815 can systemically suppress IL-6, TNFα and IL-8, while elevating epithelial expression of IL-10 and FoxP3. At the same time, EDP1815 is well tolerated with no overall difference from placebo in human trials to date. This profile could be highly relevant with respect to treating CRC.

EDP1815 works via the small intestinal axis (SINTAX): a network of anatomical and functional connections between the small intestine and the rest of the body. SINTAX links small intestinal mucosal immunology with systemic inflammation, accessible with oral medicines. EDP1815 is non-pathogenic, not genetically modified, does not colonize or persist in the gut, and does not modify the colonic microbiome. It acts locally in the gut - which is, in effect, on the outside of the body - but has pharmacodynamic activity throughout the inside of the body modulating the immune system without any systemic exposure.

EDP1815 is not systemically absorbed and there are no expected ADRs. Safety and tolerability have been indistinguishable from placebo, with no SAEs or AEs of severe intensity reported. The nature of the pharmaceutical preparation of EDP1815 ensures that there is no unwanted persistence of the drug after administration. It does not modify the colonic microbiome as assessed by 16S ribosomal RNA sequencing of stool samples from patients, and the drug is undetectable in stool

samples once dosing has ceased. Importantly, there has been no increased risk of infections observed in the phase 1 program.

The therapeutic effects of these orally delivered medicines come from their interaction with pattern recognition receptors on immune cells in the lining of the small intestine. These cells, in turn, modulate immune cells circulating throughout the body. Preclinical and clinical data provide evidence that EDP1815 is capable of modulating multiple immune pathways in humans, and therefore has the potential to become an attractive therapeutic strategy in patients with inflammatory diseases: including the abnormal host immune responses to acute viral infections.

Preclinical studies using EDP1815 have been carried out across a range of human and mouse cell in vitro assays, as well as in 5 key in vivo models, which all support the use of this agent in the treatment of immunoinflammatory diseases. In vitro, EDP1815 has been found to stimulate secretion of anti-inflammatory cytokines such as interleukin (IL)-10, IL-27, and IL-1RA, from human macrophages and dendritic cells. Furthermore, pre-clinical results have also shown that there is no effect on type 1 interferons, an important interferon for antiviral response.

In human testing EDP1815 improves psoriasis lesions (a condition due to abnormal Th17 activity) and reduces the inflammatory response to keyhole limpet hemocyanin (KLH) challenge (a Th1 driven healthy volunteer model). In addition, in ex-vivo testing EDP1815 has been shown to reduce, or normalize, the secretion of both IL-6 and IL-8, which are 2 of the key cytokines / chemokines involved in exaggerated host responses to viral infections. This confirmed the preclinical findings that EDP1815, despite being gut restricted, can modulate systemic immune biology along multiple pathways, and this unique profile and mechanism makes EDP1815 an ideal candidate for this trial.

6.1.2 Rationale for use as Therapeutic in COVID-19-related disease

An exaggerated host immune response is thought to lead to the life-threatening complications of COVID-19 infection.

Systemic cytokine interleukin (IL)-6 and chemokine IL-8 levels have been shown to be elevated in subjects hospitalised with coronaviral infections, infections with influenza A (de Jong et al, 2006; Hagau et al, 2010) and in secondary haemophagocytic lymphohistiocytosis (Wong et al 2004; Ruan et al, 2020). These exaggerated levels are pathogenic in the development of complications such as acute respiratory distress syndrome (ARDS). Although host immune response is important in the initial anti-viral response, prolonged and exaggerated immune response is associated with pulmonary complications, hospitalisation and death. A therapeutic agent that modulates multiple pathways back to a state of immune homeostasis without frank immunosuppression could offer significant clinical benefit to subjects with SARS-CoV-2 infection.

The profile of EDP1815 as an oral agent with good tolerability modulating multiple key immune pathways without abrogating them completely – that is, immune normalization rather than immune suppression – could offer significant clinical benefit to patients at risk of developing serious complications secondary to COVID-19. Its safety profile positions it as an appropriate treatment to use early in the disease course, prior to the onset of these significant symptoms or complications.

6.2 Dapagliflozin

6.2.1 Mechanism of Action

Dapagliflozin is an SGLT-2 inhibitor which blocks the predominant transporter responsible for reabsorption of glucose from glomerular filtrate back into the circulation. This leads to increased glucose excretion into the urine and to loss of sodium and water. It is licensed for control of blood glucose in patients with Type 2 diabetes at a dose of 10mg once a day.

6.2.2 Rationale for use as Therapeutic in COVID-19-related disease

Empagliflozin is structurally similar to dapagliflozin and has been shown in a rat model of pulmonary hypertension to reduce mean pulmonary artery pressure and mortality, (median survival 24 days for vehicle vs 33 days for empagliflozin) (Shinagawa et al. 2017).

Patients presenting with ARDS may have abnormalities of both cardiac and renal function including increased intra-glomerular pressure, hypertension, and fluid/sodium overload. Existing data in patients with chronic cardiac and or renal dysfunction demonstrate that SGLT2 inhibition can improve all these abnormalities (, McMurray et al 2019, Neal et al 2017, Perkovic et al 2019, Wiviott et al 2019).

6.3 Ambrisentan

6.3.1 Mechanism of Action

Ambrisentan is an orally active, propanoic acid-class, ERA selective for the endothelin A (ETA) receptor. Endothelin plays a significant role in the pathophysiology of PAH. It is approximately 4000-fold more selective for the ETA versus the ETB receptor. It is licensed for use in pulmonary arterial hypertension (PAH) at a dose of up to 10mg once a day.

6.3.2 Rationale for use as Therapeutic in COVID-19-related disease

Gattinoni et al describe the "L" phenotype of COVID-19 related respiratory failure, where there is prominent hypoxia and pulmonary shunting in the absence of marked radiographic changes consistent with ARDS. This may represent an important earlier opportunity to intervene in the progressive respiratory failure which is seen in association with COVID-19 infection. Utilising an endothelin-A (ET-A) receptor antagonist should reduce the activity of ET-1 at the ET-A receptor leading to a reduction in pulmonary shunting to hypoxic areas of the lung and blunting of inflammatory activity.

Data from cell culture suggests that interferon-gamma and TNF-alpha, inflammatory markers upregulated in ARDS drive endothelin-1 production (Shinagawa et al. 2017) Endothelin-1 levels are known to be elevated in patients with ARDS, and reduced clearance of endothelin-1 is also recognised. Persistently elevated endothelin-1 is associated with a worse outcome in patients diagnosed with ARDS (Druml et al. 1993)Pre-clinical models of endothelin antagonism in acute lung injury models support this mode of action with respect to intervention in ARDS. Tezosentan, a non-selective endothelin antagonist attenuated lung injury in

endotoxaemic sheep and LU-135252, an ET-A selective receptor antagonist improved oxygenation in an experimental pig model of acute lung injury. In a rat airway model of LPS induced lung damage, both mixed and ET-A selective antagonists were shown to reduce microvascular leakage. Interestingly, an endothelin-B (ET-B) selective antagonist showed no effect in that rat model indicating that an ET-A selective antagonist could be beneficial (Kuklin et al. 2005; Boemke et al. 2000; Hele et al. 2000).

Limited data from use of Bosentan (dual ETA/ETB receptor antagonist) in humans supports a positive effect on pulmonary hypertension and refractory hypoxaemia in a patient with ARDS and H7N9 influenza virus infection. The patient treated with Bosentan had a rapid and sustained improvement in RVSP which was followed by a gradual improvement in oxygenation (Guo et al. 2014).

Ambrisentan is a highly potent (Ki of 0.016nM), and selective ET-A receptor antagonist, with 4000 times greater affinity for the ET-A versus ET-B receptor. This has a significant potential advantage in ARDS, maintaining the positive effects of ET-1 at the ET-B receptor including vasodilatation, reduced pulmonary shunting and anti-inflammatory effects and clearance of ET-1 itself which is mediated by the ET-B receptor.

7 Trial Design

7.1 Statement of Design

Randomised, parallel arm, open-label, adaptive platform Phase 2/3 trial of potential disease modifying therapies in patients with late stage 1/stage 2 COVID-19-related disease, with a diagnosis based either on a positive assay or high suspicion of COVID-19 infection by clinical and radiological assessment. The trial will focus on novel (pre-approval) agents, novel combination of approved agents.

We propose a platform trial design which will enable evaluation of multiple treatments simultaneously under a single protocol. This design allows flexible features such as dropping treatments for safety issues or futility or adding new treatments to be tested during the course of a trial. TACTIC-E is part of the TACTIC programme of research. This protocol is aligned to the TACTIC-R study which is designed to test COVID-19 infected patients with licensed repurposed medications. Where appropriate, standard of care data from TACTIC-R (IRAS: 282213; EudraCT: 2020-001354-22) and TACTIC-E may be pooled within the TACTIC programme of COVID-19 trials.

Patients in TACTIC-E will be randomised initially in a 1:1:1 ratio across the three treatment groups. Ongoing safety analysis will allow early identification of any potential safety signals and the potential to halt that arm if the likely benefit risk ratio is no longer positive. Early futility analysis will be performed using biomarker data if feasible or clinical data if not to minimise the exposure of subjects to novel agents / combinations of agents with little chance of benefit.

Treatment with active agent will be for up to 7 days, with the option of extension to 14 days at the discretion of the PI or their delegate, if the patient is felt to be clinically responding to treatment, is tolerating treatment, and is judged to be likely to benefit from a longer treatment course. Treatment will cease when the patient is discharged from hospital or when a treatment arm is terminated on the advice of the IDMC.

7.1.1 Addition of treatment arms

The IDMC can recommend the addition of new treatment arms. These will only be considered after it is clear that recruitment rate can support additional arms and after candidate(s) have been selected from the panel discussed by the TACTIC Drug Evaluation group, and subject to formal ethical and regulatory approvals.

7.1.2 Termination of treatment arms

Treatment arms may be terminated for safety or futility on the advice of the IDMC. If a treatment arm is discontinued due to futility or safety concerns then all subjects currently being dosed will stop dosing of randomised IMP and no more subjects will be randomised to that arm.

If subjects have not received any study medication, then they can be re-randomised to one of the remaining arms.

Subjects in whom dosing has ceased will be considered to have completed active treatment, but will continue to be followed up as per the trial protocol until day 90.

7.1.3 Termination for Safety

Safety (AESI, AR, SAE, SAR, SUSAR) will be monitored in an ongoing manner and will be assessed after the first 10 subjects in each of the arms have reached day 14/discharge/primary endpoint, then after 30 subjects in each of the arms have reached day 14/discharge/primary endpoint, and then after 100 subjects in each of the arms after that (also see section5.6). The IDMC must formally confirm no reason to terminate the arm after these meetings. The timings of subsequent analyses will be determined by the IDMC. In addition, ad hoc meetings can be called at any time by the chair of the IDMC.

7.1.4 Termination for Futility

The potential for disease associated biomarkers to be used for very early futility analysis will be determined within the study. Although COVID 19 is still a very new disease, biomarkers have been identified which are associated with disease severity and risk of progression. These include:

a. CRP
b. IL-6
c. Ferritin
d. Oxygen requirements
e. D-dimer
f. Neutrophil to lymphocyte ratio
g. LDH

There are also drug specific biomarkers which are listed in section 10.5.3. The variability of a core section of these biomarkers will be assessed in the first 90 subjects (30 per group) in a treatment-allocation blind analysis of variability (standard deviation). A sub-set of these biomarkers will be selected if statistical analysis suggests they will be informative in a futility analysis using fewer than 100 subjects dosed per arm. The future biomarker interim analysis will then be formally defined including choice of biomarker, sample size and parameters to define futility, see section 15.2.2. If this is not possible, a clinical endpoint futility will be used. This analysis will be defined in more detail in the IDMC charter. If the biomarkers are not sufficiently informative, then the interim analyses based on the primary clinical efficacy endpoint will proceed as per section 15.2.3.

7.2 Participants Trial Duration

The patient will remain in the study for up to 90 (+/- 7) days for long term follow up. The trial primary endpoint will be assessed at day 14. Follow up will continue for approximately 3 months to capture secondary endpoints.

7.3 Trial Objectives

7.3.1 Primary objective

- To determine if a specific intervention reduces the composite of progression of patients with COVID-19-related disease to organ failure or death.

7.3.2 Secondary objectives

- To identify the pharmacodynamic effects of therapies on biomarkers known to be associated with progression of CRC.
- To identify pharmacodynamic effects of the therapies based on their mechanisms of action.
- To determine if a specific intervention reduces severity of disease as assessed by the 7-point ordinal scale (see Figure 8).
- To determine if a specific intervention reduces incidence of the individual endpoints of the composite.
- To assess the safety and efficacy of the different arms.
- To identify the pharmacodynamic effects of therapies on relevant biomarkers

	**Pulmonary 7-point scale**
**1**	Death
**2**	Mechanical invasive ventilation or ECMO
**3**	Non-invasive ventilation or high flow oxygen
**4**	Low flow oxygen
**5**	Hospitalised – no oxygen
**6**	Discharged; normal activities not resumed
**7**	Discharged; normal activities resumed

Figure 8. 7-point Ordinal Scale

7.3.3 Exploratory objectives

- To identify clinical or biochemical predictors of response to an intervention
- Therapy-specific markers of pharmacodynamic response
    a. EDP 1815: IL-8, TNF, IL-1β, IL-10, IL-17, IL-13
    b. Dapaglifozin and Ambrisentan: serum/plasma ET-1, TNF

7.4 Trial Outcome Measures

7.4.1 Primary outcome measure

- Time to incidence (up to Day 14) of any **<u>one</u>** of the following:
    - Death
    - Invasive mechanical ventilation
    - ECMO
    - Cardiovascular organ support (balloon pump or inotropes/ vasopressors)

  - Renal failure (Cockcroft-Gault estimated creatinine clearance <15 ml /min), haemofiltration or dialysis

7.4.2 Secondary outcome measures

- Biomarkers thought to be associated with progression of COVID-19: Ferritin, CRP, D-Dimer, neutrophil to lymphocyte ratio, LDH
- Change in clinical status as assessed on 7-point ordinal scale compared to baseline
- Time to each of the individual endpoints of the composite primary outcome measure
- Proportion of patients with adverse events of special interest in each arm
- SpO2/FiO2
- Time to $Sp0_2$ >94% on room air (excluding chronically hypoxic individuals)
- Time to first negative SARS-CoV2 PCR
- Duration of oxygen therapy (days)
- Duration of hospitalisation (days)
- All-cause mortality at day 28
- Time to clinical improvement (defined as >2 point improvement from day 1 on 7-point ordinal scale)

7.4.3 Exploratory outcome measures

- Changes in biochemical predictors and immunoinflammatory signatures of therapeutic response to inform algorithm development
- Therapy-specific markers of pharmacodynamic response
  a. EDP 1815:IL-6, IL-8, TNF, IL-1β, IL-10, IL-17, IL-13
  b. Dapaglifozin and Ambrisentan: serum/plasma ET-1, TNF

8 Selection and withdrawal of participants

8.1 Inclusion Criteria

To be included in the trial the participant must:

- be aged 18 or over
- have clinical picture strongly suggestive of COVID-19-related disease (with/without positive COVID-19 test) **AND**
  - Risk count (as defined above) >3

  OR

  - Risk count ≥3 if it includes "Radiographic severity score >3"
- be considered an appropriate subject for intervention with immunomodulatory or other disease modifying agents in the opinion of the investigator
- Is able to swallow capsules/tablets

8.2 Exclusion Criteria

8.2.1 General exclusion criteria

The presence of any of the following will preclude participant inclusion:

- Inability to supply direct informed consent from patient or from Next of Kin or Independent Healthcare Provider on behalf of patient

- Invasive mechanical ventilation at time of screening
- Contraindications to study drugs, including hypersensitivity to the active substances or any of the excipients
- Currently on any of the study investigational medicinal products
- Concurrent participation in an interventional clinical trial (observational studies allowed)
- Patient moribund at presentation or screening
- Pregnancy at screening
- Unwilling to stop breastfeeding during treatment period
- Known severe hepatic impairment (with or without cirrhosis)
- Stage 4 severe chronic kidney disease or requiring dialysis (i.e. Cockcroft Gault estimated creatinine clearance < 30 ml /min)
- Inability to swallow at screening visit
- Any medical history or clinically relevant abnormality that is deemed by the principal investigator and/or medical monitor to make the patient ineligible for inclusion because of a safety concern.

8.2.2 Drug specific exclusion criteria

8.2.3 EDP1815 Specific Exclusions

- Patient is taking a systemic immunosuppressive agent such as, but not limited to, oral steroids, methotrexate, azathioprine, ciclosporin or tacrolimus, unless these are given as part of COVID standard of care treatment.

8.2.4 Dapagliflozin and Ambrisentan Specific Exclusions

- Type 1 diabetes
- Known idiopathic pulmonary fibrosis
- Previous hospital admission with ketoacidosis
- History of symptomatic heart failure within 3 months of admission
- Sustained blood pressure below 90/60 mmHg at admission
- Metabolic acidosis defined as pH< 7.25 (or venous bicarbonate <15 mmol/l) AND ketones > 3.0 mmol/L
- Alanine transaminase and/or aspartate transaminase (ALT and/or AST) > 3 times the upper limit of normal (only one needs to be measured)

8.3 Treatment Assignment and Randomisation Number

Eligible patients will be randomised using a central web-based randomisation service initially in a 1:1:1 ratio (stratified by site) to one of the following treatment arms (each in addition to standard of care (SoC).

Arm 1: EDP1815 oral 1.6×10^{11} cells (2 capsules) twice a day for up to 7 days (may be increased to 14 days at prescriber discretion) in addition to standard of care. Patients will discontinue treatment at hospital discharge.
Arm 2: Ambrisentan 5mg ONCE a day orally for up to a maximum of 14 days and Dapagliflozin 10mg ONCE a day orally for up to a maximum of 14 days, in addition to standard of care. Patients will discontinue treatment at hospital discharge.
Arm 3: Standard of care (including approved antiviral therapies)

If a patient meets drug-specific exclusion criteria for any of the active treatment arms then they can be randomised to one of the remaining arms as long as they meet any additional drug specific inclusion/exclusion criteria.

8.4 Treatment Cessation Criteria

- Alternative clinical diagnosis appears (i.e. no longer considered to have COVID-19-related disease)
- Patient is discharged from hospital
- Progression to primary endpoint before dosing with any of the IMPs.
- Any AE indicating continued treatment is not in the best interest of the subject as assessed by the investigator
- Withdrawal of patient consent
- Unable to take randomised treatment orally
- Liver dysfunction defined as ALT or AST > 5 ULN (only 1 need be assessed) whilst on study medication for patients randomised to the Ambrisentan and Dapagliflozin treatment arm
- Metabolic acidosis (pH<7.25 or venous bicarbonate <15 mmol/l)) AND ketones > 3.0 mmol/L at any point during treatment course for patients randomised to the Ambrisentan and Dapagliflozin treatment arm
- Blood pressure persistently less than 90/60 mmHg in patients randomised to the ambrisentan and dapagliflozin treatment arm.

Patients who have been withdrawn from the trial treatment and are experiencing ongoing toxicity will be followed up until the adverse reaction comes to its conclusion. In the event of a patient being withdrawn from the trial treatment, they will continue to receive the most appropriate standard of care treatment available under the guidance of their treating clinician.

These treatment cessation patients will remain in the intention to treat analysis of the protocol and patients will continue to attend follow-up visits when they are willing and able.

8.5 Consent withdrawal

Patients may withdraw their consent to participate in the trial at any time. Withdrawal may be partial, where protocolised treatment is stopped, but allows to continue the collection of future data. Alternatively, withdrawal may be complete, in that no further trial procedures will be undertaken and no data or samples will be collected from the time of withdrawal.

However, data and samples collected up to the time of consent withdrawal will be included in the data reported for the trial. The Investigator should inform the coordination team as soon as possible and complete the consent withdrawal Case Report Form (CRF).

9 Trial Treatments

Treatment Summary

For this trial EDP1815, Dapagliflozin and Ambrisentan in the treatment arms are all considered as Investigational Medicinal Products (IMP)s conducted with a Clinical Trial Authorisation.

Any adjuvant treatments given to the patients in the standard arms are classed as Non-IMPs

Further information regarding trial treatments is contained in the Pharmacy manual.

9.1 EDP 1815

EDP1815 is a non-live pharmaceutical preparation of a single strain of *Prevotella histicola* with no genetic modification. Its mechanism of action includes the suppression of excess production of IL-6 and IL-8.

9.1.1 Legal status

EDP1815 is not licensed and is currently in clinical development in Europe and the United States of America. The proposed clinical trial is being carried out under a Clinical Trial Authorisation. The drug will only be used by the named investigators for the participants specified in this protocol, and within the trial.

9.1.2 Supply

The IMP will be supplied and distributed to sites by Evelo Biosciences Limited. Details of IMP supply can be found in the TACTIC-E pharmacy manual.

9.1.3 Packing and Labelling

The EDP1815 drug product is available as enteric coated hydroxylpropyl methylcellulose (HPMC) hard capsules in white to off-white colour. The formulation of EDP1815 consists of freeze-dried powder of P. histicola and excipients. The excipients include mannitol, magnesium stearate and colloidal silicon dioxide. Each EDP1815 capsule contains 8.0x 10^{10} cells of P histicola. EDP1815 capsules will be labelled stating the study medication and the trial name.
For further information and labelling details – refer to the Pharmacy Manual.

9.1.4 *Storage conditions*

EDP1815 should be stored in a refrigerator between 2ºC and 8ºC. Once removed from the refrigerator to room temperature (25 ºC or below), EDP1815 should be administered within 24 hours. Please refer to the Pharmacy Manual for further information about IMP storage.

The PI at each participating site or a delegated person e.g. pharmacist, is responsible for ensuring that the IMPs are stored in a secure place and under the recommended storage conditions.

9.1.5 Dosing regime and rationale

The treatment regimen for this study will be 1.6×10^{11} cells of EDP1815 in the solid dosage-in-capsule formulation given twice a day (i.e. 2 capsules, twice daily), for up to seven days. The treatment course can be extended to 14 days at the discretion of the principal investigator or one of their designees – if the patient is felt to be responding to treatment. The treatment will be stopped prior to this time if/when the patient is discharged from hospital.

The dose range of EDP1815 tested in humans to date (1.6×10^{11} cells to 8.0×10^{11} cells in EDP1815-101) was based on allometric scaling from the pre-clinical *in vivo* experimental data. Both doses had clear effects on IL-6 and IL-8 based on lipopolysaccharide (LPS) stimulation of whole blood samples taken at baseline and after a course of daily administration of EDP1815. The study included patients with mild to moderate psoriasis, and improvements in their skin condition were also demonstrated. There was no clear difference between the 2 dose levels tested on IL-6 and IL-8 and so the lower dose has been selected for this study.

A twice daily (bd) dosing regimen has been selected to maximise the speed of response. EDP1815 works via direct interaction with immune cells in the epithelium of the upper small intestine. A twice a day regimen doubles the duration of exposure of the microbes to the immune cells in the upper small intestine per 24 hours and will increase the speed of response. The total daily dose is less than half the maximum daily dose tested for 28 days in EDP1815-101.

9.1.6 Maximum duration of treatment of a participant

14 days

9.1.7 Administration

EDP1815 will be administered orally twice daily for up to 14 days. EDP1815 should be taken on an empty stomach i.e. one hour before or two hours after food. The capsules should be swallowed whole with water and not chewed or opened. There should be at least a two-hour window between the twice-daily dosages.

9.1.8 Contraindications, Medicines Interactions

There are no contraindications to treatment. There are no prohibited concomitant medications and no anticipated drug-drug interactions. Of note, no significant inhibition or induction of any of the CYP450 enzymes were observed *in vitro* after EDP1815 treatment.

9.1.9 Dose modifications

Dose modifications are not planned for this trial.

9.1.10 Side effects

EDP1815 is not systemically absorbed and there are no expected ADRs. Safety and tolerability have been indistinguishable from placebo, with no SAEs or AEs of severe intensity reported. The non-replicating nature of the pharmaceutical preparation of EDP1815 ensures that there is no unwanted persistence of the drug after administration. It does not modify the colonic microbiome as assessed by 16S ribosomal RNA sequencing of stool samples from patients, and the drug is

undetectable in stool samples once dosing has ceased. Importantly, there has been no increased risk of infections.

9.2 Dapagliflozin

Dapagliflozin is a sodium-glucose co-transporter 2 (SGLT-2) inhibitor.
It lowers blood sugar by causing the kidneys to get rid of more glucose in the urine.

9.2.1 Legal status

Dapagliflozin is licensed for use in the UK for treatment of Type II diabetes. Since this trial is evaluating Dapagliflozin in an unlicensed indication, it is being carried out under a Clinical Trial Authorisation (CTA). It is therefore only to be used by the named Investigators for the participants specified in this protocol, and within the trial.

9.2.2 Supply

Dapagliflozin will be supplied by Astrazeneca free of charge for this trial. The supply should be ring-fenced at the participating site for this trial in a separate area to non-trial products and records retained in the Pharmacy Site File noting the location of the storage. It will be distributed directly to site following confirmation that all necessary regulatory and ethical approvals are in place. Please see the Pharmacy Manual for further information.

9.2.3 Packing and Labelling

Dapagliflozin is presented as a biconvex, yellow film coated tablet with '10' engraved on one side and '1428' engraved in the other side. Commercially available packs will be provided to sites. IMP label templates will be provided to participating sites for local labelling by the respective Pharmacy departments.

9.2.4 Storage conditions

Dapagliflozin must be kept in a secure place under appropriate storage conditions as per SmPC. The IMP should be stored in an area free of environmental extremes and a secured area to prevent unauthorised access. The storage area will be temperature controlled (i.e. air-conditioning and heating controls available) but temperature readings will not be monitored unless there is cause for concern.

9.2.5 Maximum duration of treatment of a participant

Participants will receive the IMP for up to 14 days

9.2.6 Dose

Dapagliflozin 10mg should be taken orally once daily with or without food. Tablets are to be swallowed whole.

9.2.7 Administration

Oral

9.2.8 Dose modifications

No dose modifications are required in this study.

9.2.9 Side effects

The most frequently reported side-effects across the Type 2 diabetes study program were recurrent genital infections. Other side effects of most relevance to the COVID-19 population include diabetic ketoacidosis and volume depletion. In the dapagliflozin cardiovascular outcomes study, with a median exposure time of 48 months, events of DKA were reported in 27 patients in the dapagliflozin 10 mg group and 12 patients in the placebo group. The events occurred evenly distributed over the study period. Of the 27 patients with DKA events in the dapagliflozin group, 22 had concomitant insulin treatment at the time of the event.

In the 13-study safety pool, reactions suggestive of volume depletion (including, reports of dehydration, hypovolaemia or hypotension) were reported in 1.1% and 0.7% of subjects who received dapagliflozin 10 mg and placebo, respectively; serious reactions occurred in < 0.2% of subjects balanced between dapagliflozin 10 mg and placebo (see section 4.4).

In the dapagliflozin cardiovascular outcomes study, the numbers of patients with events suggestive of volume depletion were balanced between treatment groups: 213 (2.5%) and 207 (2.4%) in the dapagliflozin and placebo groups, respectively. Serious adverse events were reported in 81 (0.9%) and 70 (0.8%) in the dapagliflozin and placebo group, respectively. Events were generally balanced between treatment groups across subgroups of age, diuretic use, blood pressure and ACE-I/ARB use. In patients with eGFR < 60 mL/min/1.73 m2 at baseline, there were 19 events of serious adverse events suggestive of volume depletion in the dapagliflozin group and 13 events in the placebo group.
All known reactions are summarised in section 4.8 of dapagliflozin SmPC approved by the MHRA for use in this trial.

9.3 Ambrisentan

9.3.1 Description

Ambrisentan is an endothelin receptor antagonist, and is selective for the type A endothelin receptor (ETA).

9.3.2 Legal status

Ambrisentan was approved by the U.S. Food and Drug Administration (FDA) and the European Medicines Agency (EMA) and indicated for the treatment of pulmonary arterial hypertension.

9.3.3 Supply

Ambrisentan is available commercially and will be obtained locally by the investigating sites.

9.3.4 Labelling

IMP label templates will be provided to participating sites for local labelling by the respective Pharmacy departments.

9.3.5 Storage conditions

Ambrisentan must be kept in a secure place under appropriate storage conditions as per its SmPC. The IMP should be stored in an area free of environmental extremes and a secured area to prevent unauthorised access. The storage area will be temperature controlled (i.e. air-conditioning and heating controls available) but temperature readings will not be monitored unless there is cause for concern

9.3.6 Maximum duration of treatment of a participant

Ambrisentan should be taken for a maximum of 14 days

9.3.7 Dose

Ambrisentan is administered at 5mg dose once a day. The tablets can be taken with or without food and should be swallowed whole and not split, crushed or chewed.

9.3.8 Dose modifications

No dose modification is necessary for this study

9.3.9 Side effects

Peripheral oedema has been observed with ERAs including ambrisentan. Most cases of peripheral oedema in clinical studies with ambrisentan were mild to moderate in severity, although it may occur with greater frequency and severity in patients ≥65 years. Peripheral oedema was reported more frequently with 10 mg ambrisentan in short-term clinical studies.

Post-marketing reports of fluid retention occurring within weeks after starting ambrisentan have been received and, in some cases, have required intervention with a diuretic or hospitalisation for fluid management or decompensated heart failure. If patients have pre-existing fluid overload, this should be managed as clinically appropriate prior to starting ambrisentan.

The incidence of peripheral oedema was increased when ambrisentan was dosed in combination with tadalafil (45% adverse event frequency), compared to the incidence of peripheral oedema when ambrisentan and tadalafil were given as monotherapy (38% and 28%, respectively). The occurrence of peripheral oedema was highest within the first month of treatment.

Liver aminotransferases (ALT and AST) should be evaluated prior to initiation of ambrisentan and treatment should not be initiated in patients with baseline values of ALT and/or AST >3xULN.

If patients develop sustained, unexplained, clinically significant ALT and/or AST elevation, or if ALT and/or AST elevation is accompanied by signs or

symptoms of hepatic injury (e.g. jaundice), ambrisentan therapy should be discontinued.

In patients without clinical symptoms of hepatic injury or of jaundice, re-initiation of ambrisentan may be considered following resolution of hepatic enzyme abnormalities. The advice of a hepatologist is recommended.

Shortness of breath and hypotension are also commonly reported in patients taking ambrisentan therapy, as is cardiac failure. Cardiac failure was reported more commonly in patients who also had peripheral oedema.
All known reactions are summarised in section 4.8 of Ambrisentan SmPC approved by the MHRA for use in this trial.

9.3.10 Additional information

It is thought that utilising a maximum dose of 5mg of ambrisentan in this study combined with dapagliflozin will significantly mitigate any effects of fluid retention associated with ambrisentan use.

9.4 Active comparator products

EDP1815 + standard of care will be actively compared to standard of care alone.

Dapagliflozin and Ambrisentan combination therapy + standard of care will be actively compared to standard of care alone.

No comparisons will be made between active arms in this platform trial.

Standard of Care:
Participants receiving standard of care can receive any approved standard therapy (which may include approved antiviral therapies), at the discretion of the Investigator, and on discussion with the participant.

9.4.1 Missed or replacement doses

If vomiting occurs shortly after taking trial medication, the dose should not be replaced. The next scheduled dose should be taken at the normal time. If any doses are omitted for any reason, these should not be made up later in the day, or at the end of the dosing period.

9.5 Accountability and dispensing

The IMPs should only be used as directed in this protocol.

9.5.1 Pharmacy Responsibilities:

All pharmacy aspects of the trial at a participating site are the responsibility of the Principal Investigator (PI) who will delegate this responsibility to the local pharmacist, or other appropriately qualified personnel. This delegation of duties must be recorded on the delegation log. The PI or a delegated individual (e.g. the trial pharmacist), will ensure that the trial medications are stored and dispensed in accordance with local practice, applicable regulatory requirements and trial-specific prescriptions.

The PI and/or delegated staff are fully responsible for the IMP at site. Dispensing of medication will be delegated to the hospital pharmacy.

9.5.2 Dispensing

Dispensing should occur by the pharmacy at the participating site in accordance with the trial-specific prescription or as per local site specific procedures. A prescription template will be provided, although sites will be permitted to use their own clinical trial template prescription if suitable

Refer to the Pharmacy manual for details regarding ordering, stock levels, temperature monitoring and quarantine procedures.

9.5.3 Drug accountability

The sites will be required to keep accountability records detailing all IMP shipments if applicable, storage and dispensing. Further details regarding the drug accountability can be found in the Pharmacy manual

9.5.4 Returns and destruction and treatment compliance

The nature of this study means that the return of IMPs dispensed to hospital wards may not be possible and returns are not, therefore, a formal sponsor requirement. As all IMPs are dosed as inpatients, evidence of dosing may be obtained from hospital administration records.

If returns are made, destruction according to local procedures is permitted, after successful local reconciliation and documentation. No further sponsor approval is required.

Refer to Pharmacy manual for further details.

10 Procedures and assessments

Trial assessments and procedures will be performed by suitably qualified and delegated trial personnel as described in the TPM.

10.1 Participant identification

Potential patients will be identified by an attending clinician upon arrival to the participating hospital if they are strongly suspected to be or are COVID-19 positive. Suitable patients will be approached and referred to the research team if appropriate. This may be achieved by reviewing inpatient medical notes (by a

member of the clinical team or suitably qualified, delegated team member) or by discussion with clinical teams regarding their inpatients. Patients will be referred to the research team if they are interested in participating in this clinical trial.

There will be study advertisements placed in clinical areas, web-based (online/generic Trust emails/newsletters) and social media platforms. Members of the research team who are also direct care team members will monitor admissions, electronic track boards in the emergency department and admissions ward and may receive COVID result alerts to identify potential participants.

Once contact has been made with the patient, the research team will outline and explain the aims of the trial. A copy of the Patient Information Sheet will then be given to the patient who will have the opportunity to consider the information and discuss the trial with the trial staff and raise any queries before consenting to participate in the trial.

10.2 Consent

Informed consent should be obtained from each patient before enrolment into the study. In line with other urgent COVID -19 trials such as RECOVERY if the patient lacks capacity to give consent due to the severity of their medical condition (e.g. acute respiratory failure or delirium), then consent may be obtained from a relative acting as the patient's legally designated representative. Further consent will then be sought with the patient if they recover sufficiently.

Due to the poor outcomes in COVID-19 patients who require ventilation (>50% mortality in one cohort), patients who lack capacity to consent due to severe disease (e.g. severe hypoxia), and for whom a relative to act as the legally designated representative is not immediately available, randomisation and consequent treatment will proceed with consent provided by a treating clinician (independent of the clinician seeking to enrol the patient) who will act as the legally designated representative. Consent will then be obtained from the patient's personal legally designated representative (or directly from the patient if they recover promptly) at the earliest opportunity.

10.3 Screening evaluation

10.3.1 Screening Assessments (Day -2 to Day -1)

Trial specific assessments will only be conducted after written informed consent has been provided. Due to the urgent nature of the study, it is anticipated that screening and baseline assessments will occur on the same day for most participants.

The results of screening tests must be available before randomisation and IMP dosing. Baseline tests will be done but results do not need to be available before dosing.

The screening/baseline visit will take place on inpatient wards/an appropriate space in the hospital. The following procedures will be performed at this visit:

Screening procedures:

- Consent
- Review of medical history and whole medical record

- Clinical examination
- Medication review
- Full blood count (including differential white cell count)*
- Calculated Cockcroft Gault creatinine clearance*
- ALT* OR AST* (only 1 required)
- Venous blood gas
- Blood ketone (point of care)
- CRP*
- Chest X-ray**
- Pregnancy test (blood OR urine)***
- Eligibility check of inclusion/exclusion criteria

*The results of these tests acquired up to 48hr before consent may be used to complete the screening and eligibility process.

** When available, information from a chest X-ray, performed as part of routine care, will be used to calculate the radiological severity score, if the patient meets the Risk Count this they may still enter the trial. Chest X-rays will NOT be performed as part of this protocol.

*** Pregnancy test will not be performed on post-menopausal women (for the purposes of this trial, postmenopausal is defined as being amenorrhoeic for greater than 2 years with appropriate clinical profile, e.g. age appropriate, history of vasomotor symptoms)

Eligible subjects should be randomised as soon as possible after eligibility is confirmed, with dosing occurring on the same day where possible.

10.3.2 Participant Randomisation

All patients screened for the trial will be assigned a unique participant ID number All screening tests must be available and checked by the delegated trial doctor before randomisation. The PI or delegate must sign the CRF to confirm eligibility after the screening process has been completed.

Suitable participants will be subsequently randomised at the participants' research site using a web-based online system. Randomisation notifications will be sent via email to research staff at the participating site including pharmacy as well as to the trial coordinator. Notifications will include information on drug allocation for the randomised patient. Further details can be found in the TPM.

10.4 Baseline Assessments (Day -2 to Day -1)

The following assessments will be undertaken either at the time of screening or soon after randomisation and the data points to be recorded at baseline are:

- Days since onset of symptoms
- Demographics and anthropomorphic data (age, height, weight etc)
- Vital signs (from medical records for example blood pressure, heart rate, oxygen saturations, temperature: as defined in TPM)
- Oxygen therapy status (e.g. FiO2, SaO2)

- Document current position on 7-point ordinal scale
- COVID-19 RTPCR result (if available)
- Extraction of clinical data from medical records (e.g. other bloods, radiology, etc.)
- Biomarkers (see section 10.5.3)
- Optional research blood samples/venous endothelial cells (section 10.5.4)

After randomisation all patients will receive EDP1815; or combination Ambrisentan + Dapagliflozin; or standard care according to the randomisation schedule, plus all ongoing standard of care treatment.

10.5 Trial assessments

Trial visits will be conducted at the trial sites.

10.5.1 Timing of in hospital assessments

Daily assessments will be performed on days 2-14 (or until discharge, whichever comes sooner):

- Vital signs (from medical records for example blood pressure, heart rate, oxygen saturations, temperature: as defined in TPM)
- Oxygen therapy status (e.g. FiO2)
- Document current position on 7-point ordinal scale
- Obtain COVID-19 RTPCR results (if available)
- For patients randomised to ambrisentan and dapagliflozin assessment of acid base status (Ketoacidosis defined as pH<7.25 or venous bicarbonate <15 mmol/l) AND blood ketones >3.0 mmol/l) should occur at least daily
- Review of adverse events

Additional assessments (Days 2, 6 and 14) ± 2 days

- Full blood count (including differential white cell count)*
- Calculated Cockcroft Gault creatinine clearance*
- ALT* or AST*
- CRP*
- Biomarkers – Ferritin, DDimer, LDH (section 10.5.3)
- Optional research blood sampling/venous endothelial cells (section 10.5.4)
- Routine retrieval of clinical data

*The results of these tests acquired within a 96 hour window may be used.
The Day 14 or discharge assessments will be done where feasible.

10.5.2 Further assessments and end of trial visit

These visits (in person or by telephone consultation with participant or family member) will be held approximately 28 (+/- 7 days) days and 90 (+/- 7 days) days after the baseline visit.

Assessments will include the following:

- Discharge status
- Return to normal function status (numeric rating scale 0-10)
- Urine pregnancy test
- Mortality status
- Adverse event reporting

At selected sites, optional research samples may be taken for assays of potential biomarkers of response including but not confined to immunological and genomic transcriptomic and cellular analyses for future analysis. Venous endothelial cells may also be collected to look at the effect of COVID-19 on endothelial cells function and the impact of IMPs. Data from clinically performed tests such as echocardiograms, chest X-rays or lung function tests may be retrieved if available.

10.5.3 Biomarker Assessments

Disease relevant biomarker data will be collected across all arms of the trials, and will be assessed for its potential to be used as an early stopping criteria for futility. If the biomarker data is established as informative / potentially informative then this data will be used in an initial futility analysis.

Biomarkers to be collected at baseline, day 2, 6, and 14/discharge/primary endpoint are:

- CRP
- Neutrophil:Lymphocyte ratio
- Ferritin
- D-dimer
- LDH

Due to the different mechanisms of action of the active interventions, the following relevant specific biomarker data will also be included in the analysis for each agent where possible. This may not be done depending on initial or subsequent reviews or where it may not be practicable:

EDP1815 (optional):

- TNF,
- IL6,
- IL8,
- IL1b,
- IL10,
- IL17,
- IL13 and others

Ambrisentan & Dapagliflozin (optional):

- ET1, TNF and IL6 and others may be carried out at sites with capability to process and store these samples. Additional samples may be stored for future analyses.

Biomarkers will be assessed where possible and inability to collect all or some at a particular time point does not exclude the patient from participating.

10.5.4 Research Blood Sampling

Additional samples may be taken at selected sites with the capability and capacity for testing and storage. These samples will be taken at baseline, day 2, day 6, and day 14/discharge. Samples will be stored for assays of additional biomarkers of response; including but not confined to immunological and genomic transcriptomic and cellular analyses for future analysis.

10.6 Schedule of Assessments

Time and Events table for data collection during TACTIC-E Study. All dosing and other assessments will be performed/retrieved from the medical record if the patient is a current inpatient and will be discontinued (apart from follow-up) if the patient is deemed clinically fit for discharge.

**Data**	**Screening (Day -2 to Day -1)^**[c]	**Baseline (Day-2 to Day -1)^**	**D1 ^**	**D2 ***	**D3**	**D4**	**D5**	**D6 ***	**D7**	**D8**	**D9**	**D10**	**D11**	**D12**	**D13**	**Optional D14* or discharge date***	**Follow up (~28 days and 90 days)***
Informed consent	x																
Eligibility criteria	x																
Medical history	x																
Physical examination	x																
Vital signs[#]		x	x	x	x	x	x	x	x	X	x	x	x	x	x	x	
Oxygen therapy status[#]		x	x	x	x	x	x	x	x	x	x	x	x	x	x	x	
Medication review	x			x				x								x	
Clinically indicated blood tests retrieved from medical record: creatinine, ALT or AST [#*]	x [c]			x				x								x	
Routine retrieval and review of relevant clinical data[#]	x	x		x				x								x	x*
Chest X-ray review for risk score (extracted from medical record, not mandated as part of trial protocol)[#d]	x																x[e]
Pregnancy test (blood/urine)	x																x
Day since onset of symptoms		x		x				x								x	x
Demographics and anthropomorphic data		x															
7-point ordinal scale		x	x	x	x	x	x	x	x	x	x	x	x	x	x	x	
COVID-19 RTPCR (result may not be available prior		x														x	

to dosing)[#]																	
spO2 and FiO2		x		x				x								x	
Biomarker tests[#*] (section 10.5.3) Protocol mandated: FBC (for neutrophil:lymphocyte ratio), CRP, Ferritin, DDimer, LDH, and optionally (where sites are capable) via a plasma store: IL-6, IL8. IL1β, IL-10, IL-17, IL-13, Endothelin-1, TNF		x		x				x								x	
Research blood sampling/venous endothelial cells [a,b]		x		x				x								x	x
Venous blood gas (pH) or venous bicarbonate: Screening for all patients & thereafter for Ambrisentan + Dapagliflozin arm only	x		x	x	x	x	x	x	x	x	x	x	x	x	x	x	
Blood ketone POC: Screening for all patients & thereafter for Ambrisentan + Dapagliflozin arm only	x		x	x	x	x	x	x	x	x	x	x	x	x	x	x	
Review of adverse events			x	x	x	x	x	x	x	x	x	x	x	x	x	x	x
Discharge status																	x
Return to normal function status (ECOG and MRC Dyspnoea scores)																	x
Mortality status																	x
EDP1815 arm only – drug administration			x	x	x	x	x	x	x	x[e]	x[e]	x[e]	x[e]	x[e]	x[e]	x[e]	
Ambrisentan and Dapagliflozin arm only – drug administration			x	x	x	x	x	x	x	x[e]	x[e]	x[e]	x[e]	x[e]	x[e]	x[e]	

[a] Samples could be stored for assays of additional biomarkers of response; including but not confined to immunological and genomic transcriptomic and cellular analyses for future analysis
[b] Research sampling is optional where units have capability – not mandatory
[c] The results of these tests acquired up to 48hr before consent may be used to complete the screening and eligibility process.
[d] A clinically indicated chest X-ray will be reviewed from the patient's medical record to perform the risk score. This is not a trial mandated procedure.
[e] Treatment can continue beyond 7 days to day 14, at the discretion of the PI or his delegate, if the patient is felt to be clinically responding to treatment, is tolerating treatment, and is judged to be likely to benefit from a longer treatment course. Treatment will cease when the patient is discharged from hospital (even if this occurs before Day 7).

* For D2, D6 and D14 (+/-2 days): The results of FBC, Cockcroft Gault Creat. Clearance, ALT/AST and CRP acquired within a **96 hour window** may be used
^ Can be performed on the same day
# Results will be extracted from the patient record where available

10.7 End of Trial Participation

The patient's participation in the trial will end once they have completed their Day 90 follow-up visit or withdrawn their consent for the trial. However, all on-going ARs must continue to be followed after their participation has ceased until they have been resolved. Patients will return to normal standard of care when the treatment period has terminated, if the treatment arm they are on is terminated or on discharge.

10.8 Trial restrictions

Sexually active women of childbearing potential are required to use adequate contraception for the duration of the 90-day trial following completion of the last treatment. This includes:

- Intrauterine Device (IUD)
- Hormonal based contraception (pill, contraceptive injection or implant etc)
- Barrier contraception (condom and occlusive cap e.g. diaphragm or cervical cap with spermicide) alone is not considered adequate contraception for this study
- True abstinence (where this is in accordance with the participants' preferred and usual lifestyle)

Sexually active men are required to use adequate contraception for the entire duration of the trial and, if on either active arm, for 90 days following completion of the last treatment. This includes:

- Barrier contraception (condom and spermicide) even if female partner(s) are using another method of contraception or are already pregnant (also to protect male partners from exposure to the trial IMPs etc)
- True abstinence (where this is in accordance with the participants' preferred and usual lifestyle)

11 Assessment of Safety

11.1 Definitions

11.1.1 Adverse event (AE)

Any untoward medical occurrence in a participant or clinical trial participant administered a medicinal product and which does not necessarily have a causal relationship with this treatment.

An adverse event can therefore be any unfavourable and unintended sign (including an abnormal laboratory finding), symptom, or disease temporally associated with the use of an investigational medicinal product, whether or not considered related to the investigational medicinal product.

Please note: Recording of all adverse events must start from the point of Informed Consent regardless of whether a participant has yet received a medicinal product.

11.1.2 Adverse reaction to an investigational medicinal product (AR)

All untoward and unintended responses to an investigational medicinal product related to any dose administered. All adverse events judged by either the reporting investigator or the sponsor as having a reasonable causal relationship to a medicinal product qualify as adverse reactions. The expression reasonable causal relationship means to convey in general that there is evidence or argument to suggest a causal relationship.

11.1.3 Unexpected adverse reaction

An adverse reaction, the nature, or severity of which is not consistent with the applicable reference safety information (RSI) (e.g. investigator's brochure for an unapproved investigational product or summary of product characteristics (SmPC) for an authorised product).

When the outcome of the adverse reaction is not consistent with the applicable RSI this adverse reaction should be considered as unexpected.

The term “severe” is often used to describe the intensity (severity) of a specific event. This is not the same as “serious,” which is based on participant /event outcome or action criteria.

11.1.4 Serious adverse event or serious adverse reaction (SAE / SAR)

Any untoward medical occurrence that at any dose:

- results in death
- is life-threatening
- requires hospitalisation or prolongation of existing inpatient’s hospitalisation
- results in persistent or significant disability or incapacity
- is a congenital anomaly or birth defect.
- is an important medical event - Some medical events may jeopardise the participant or may require an intervention to prevent one of the above characteristics/ consequences. Such events (hereinafter referred to as ‘important medical events’) should also be considered as ‘serious’

Life-threatening in the definition of a serious adverse event or serious adverse

reaction refers to an event in which the participant was at risk of death at the time of event; it does not refer to an event which hypothetically might have caused death if it were more severe.

11.1.5 Suspected Unexpected Serious Adverse Reaction (SUSAR)

A serious adverse reaction, the nature and severity of which is not consistent with the information set out in the Reference Safety Information

11.1.6 Reference Safety Information (RSI)

A list of medical events that defines which reactions are expected for the IMP within a given trial and thus determining which Serious Adverse Reactions (SARs) require expedited reporting.

The RSI is contained in a clearly identified section of the Summary of Product Characteristics (SmPC) or the Investigator's Brochure (IB)

For this trial the Reference Safety Information is:
Section 4.8 of the SmPC for:
Forxiga (Dapagliflozin), dated 02 Jan 2020
Volibris (Ambrisentan , dated 12 Nov 2018

Section 8 of EDP1815 Investigator's Brochure Version 2.1 dated 28 January 2020.
As studies of EDP1815 are clinically ongoing, no expected SARs are listed in the RSI section of the current IB. As such, all SARs related to EDP1815 only in this trial will be reported as SUSARs.

11.2 Expected Adverse Reactions/Serious Adverse Reactions (AR /SARs)

All expected Adverse Reactions are listed in the latest MHRA approved version of the RSI as specified in section 11.1.6. This must be used when making a determination as to the expectedness of the adverse reaction. If the adverse reaction meets the criteria for seriousness, this must be reported as per section 11.5.

11.3 Evaluation of adverse events

The Sponsor expects that adverse events are recorded from the point of Informed Consent regardless of whether a participant has yet received a medicinal product. Individual adverse events should be evaluated by the investigator. This includes the evaluation of its seriousness, and any relationship between the investigational medicinal product(s) and/or concomitant therapy and the adverse event (causality).

11.3.1 Assessment of seriousness

Seriousness is assessed against the criteria in section 11.1.4. This defines whether the event is an adverse event, serious adverse event or a serious adverse reaction

11.3.2 Assessment of causality

Definitely: A causal relationship is clinically/biologically certain. **This is therefore an Adverse Reaction**

Probable: A causal relationship is clinically / biologically highly plausible and there is a plausible time sequence between onset of the AE and administration of

the investigational medicinal product and there is a reasonable response on withdrawal. **This is therefore an Adverse Reaction.**

Possible: A causal relationship is clinically / biologically plausible and there is a plausible time sequence between onset of the AE and administration of the investigational medicinal product. **This is therefore an Adverse Reaction.**

Unlikely: A causal relation is improbable and another documented cause of the AE is most plausible. **This is therefore an Adverse Event.**

Unrelated: A causal relationship can be definitely excluded and another documented cause of the AE is most plausible. **This is therefore an Adverse Event.**

Unlikely and Unrelated causalities are considered NOT to be trial drug related
Definitely, Probable and Possible causalities are considered to be trial drug related

A pre-existing condition must not be recorded as an AE or reported as an SAE unless the condition worsens during the trial and meets the criteria for reporting or recording in the appropriate section of the CRF as specified in section 11.1.1 and 11.3.

11.3.3 Clinical assessment of severity

Mild: The participant is aware of the event or symptom, but the event or symptom is easily tolerated

Moderate: The participant experiences sufficient discomfort to interfere with or reduce his or her usual level of activity

Severe: Significant impairment of functioning; the subject is unable to carry out usual activities and / or the participant's life is at risk from the event.

11.3.4 Recording of adverse events

Adverse events will be recorded in medical notes only. Due to the underlying clinical condition of the trial population it is not practicable to report all adverse events in this trial and it is thought that excessive safety reporting may detract from the main objectives of the trial. Rather, only AEs of special interest (AESI) should be reported as detailed in section 11.7.

Adverse reactions should be recorded in the medical notes and the appropriate of the CRF and/or AR log. Serious Adverse Events will be recorded and reported in this trial. Serious Adverse Reactions should be reported to the sponsor as detailed in section 11.4.

11.4 Recording and Reporting SAEs and SARs

All serious adverse events will be recorded in the trial data collection tools. All will be reported to the Chief Investigator using the trial specific SAE form within 24 hours of their awareness of the event.

The Chief Investigator is responsible for ensuring that the assessment of all SAEs for relatedness and expectedness is completed and the onward notification of all SARs Sponsor immediately but not more than 24 hours of first notification. The

sponsor has to keep detailed records of all SARs reported to them by the trial team. All SAEs/SARs may be reported by the trial team to the relevant pharmaceutical company, in line with contractual requirements.

The Chief Investigator is also responsible for prompt reporting of all Serious Adverse Reactions to the competent authority (e.g. MHRA) of each concerned Member State if they could:

- adversely affect the health of participants
- impact on the conduct of the trial
- alter the risk to benefit ratio of the trial
- alter the competent authority's authorisation to continue the trial in accordance with Directive 2001/20/EC

The completed SAE/SAR form must be emailed to cctupharmacovigilance@addenbrookes.nhs.uk and cambs.cardiovscular@nhs.net . Additionally SAE/SARs for the EDP1815 arm should be sent to medpace-safetynotification@medpace.com and SAE/SARs for the Dapaglifozin/Ambrisentan arm should ALSO be sent to AEMailboxClinicalTrialTCS@astrazeneca.com. Details of where to report the SAE/SARs can be found on the 'TACTIC-E' SAE/SAR form and the front cover of the protocol.

11.5 Reporting of Suspected Unexpected Serious Adverse Reactions (SUSARs)

All suspected adverse reactions related to an investigational medicinal product (the tested IMP and comparators) which occur in the concerned trial, and that are both unexpected and serious (SUSARs) are subject to expedited reporting. Please see section 11.1.6 for the Reference Safety Information to be used in this trial.

11.5.1 Who should report and whom to report to?

The Sponsor delegates the responsibility of notification of SUSARs to the Chief Investigator. The Chief Investigator must report all the relevant safety information previously described, to the:

- Sponsor
- Competent authorities in the concerned member states (eg MHRA)
- Ethics Committee in the concerned member states

The Chief Investigator shall inform all investigators concerned of relevant information about SUSARs that could adversely affect the safety of participants. SUSARs may also be reported to the license holder if requested.

11.5.2 When to report?

11.5.3 Fatal or life-threatening SUSARs

The CI must inform the Sponsor of any fatal SUSAR immediately but within 24 hours of the site investigator awareness of the event. The MHRA and Ethics Committee must be notified as soon as possible but no later than **7 calendar days** after the trial team and Sponsor has first knowledge of the minimum criteria for expedited reporting.

In each case relevant follow-up information should be sought and a report completed as soon as possible. It should be communicated to all parties within an additional **8 calendar days**.

11.5.4 Non-fatal and non-life-threatening SUSARs

All other SUSARs and safety issues must be reported to the Sponsor immediately but within 24 hours of the site investigator awareness of the event . The MHRA and Ethics Committee must be notified as soon as possible but no later than **15 calendar days** after first knowledge of the minimum criteria for expedited reporting. Further relevant follow-up information should be given as soon as possible.

11.5.5 Method and timelines of SUSAR reporting

11.5.6 Minimum criteria for initial expedited reporting of SUSARs

Information on the final description and evaluation of an adverse reaction report may not be available within the required time frames for reporting. For regulatory purposes, initial expedited reports should be submitted within the time limits as soon as the minimum following criteria are met:
a) a suspected investigational medicinal product
b) an identifiable participant (e.g. trial participant code number)
c) an adverse event assessed as serious and unexpected, and for which there is a reasonable suspected causal relationship
d) an identifiable reporting source

and, when available and applicable:

- an unique clinical trial identification (EudraCT number or in case of non-European Community trials the sponsor's trial protocol code number)
- an unique case identification (i.e. sponsor's case identification number)

11.5.7 Follow-up reports of SUSARs

In case of incomplete information at the time of initial reporting, all the appropriate information for an adequate analysis of causality should be actively sought from the reporter or other available sources. Further available relevant information should be reported as follow-up reports.
In certain cases, it may be appropriate to conduct follow-up of the long-term outcome of a particular reaction.

11.5.8 Format of the SUSARs reports

Electronic reporting is the expected method for expedited reporting of SUSARs to the competent authority. The format and content as defined by the competent authority should be adhered to.

11.6 Pregnancy Reporting

All pregnancies within the trial (either the trial participant or the participant’s partner) should be reported to the Chief Investigator and the Sponsor using the relevant Pregnancy Reporting Form within 24 hours of awareness. Pregnancies must be reported for all patients for the duration of their trial participation until the 3 month follow-up visit.

All pregnancies will be reported by the trial team to the relevant pharmaceutical company in line with the contractual requirements.

Pregnancy is not considered an AE unless a negative or consequential outcome is recorded for the mother or child/fetus. If the outcome meets the serious criteria, this would be considered an SAE.

11.7 Adverse events of special interest (AESIs)

For this trial, the following adverse events will be recorded as AESIs:

- Diabetic ketoacidosis in those patients on Ambrisentan & Dapagliflozin
- New peripheral oedema in those patients on Ambrisentan & Dapagliflozin arm

Each Principal Investigator must report all AESIs to the CI using the CRF in a timely manner. If the AESI is deemed to be serious, then the reporting procedure for an SAE should be followed as detailed in section 11.5

12 Toxicity – Emergency Procedures

In the event of an acute hypersensitivity to any of the IMPs, supportive care will be given to the patient according to local clinical procedures.

13 Evaluation of Results (Definitions and response/evaluation of outcome measures)

Please refer to section 7.5 Trial Outcome Measures

13.1 Response criteria

Please refer to section 7.5 Trial Outcome Measures

14 Storage and Analysis of Samples

Research samples that are to be analysed at a later stage are to be stored in an appropriate manner at the local site. Only the research team will have access to these samples and analyse when logistics permit.

15 Statistics

15.1 Statistical methods

The primary endpoint will compare the experimental treatments to control using Cox proportional hazards, adjusting for important baseline prognostic predictors (age, gender, ethnicity, radiological severity score, underlying health condition, neutrophils, CRP, and recruiting site). Estimates, 95% confidence intervals and p-values will be provided for the treatment effects on the hazard ratio (HR) scale. The main analyses will be conducted by the trial statistician following the intention-to-treat principle.

Secondary endpoints will be analysed using a similar regression methodology, as suitable for the nature of the endpoint (binary, categorical, continuous, time-to-event).

15.2 Interim analyses

15.2.1 Ongoing Safety Monitoring

A safety subgroup of the IDMC will review safety data (i.e. AESI, AR, SAE, SAR, SUSARs) after the first 10 subjects have reached Day 14/discharge/primary endpoint in each of the arms. This will be repeated after 30 per arm have reached Day 14/discharge/primary endpoint in each of the arms and again at 100 subjects per arm. The timings of subsequent analyses will be determined by the IDMC. Further analysis described in sections 15.2.2 and 15.3.3. These blinded data reviews will be by cohort and can be unblinded if required. A decision to stop a treatment arm can be made by this subgroup if there is any concern regarding safety of an active arm. This will provide ongoing regular safety monitoring of all cohorts.

15.2.2 Early Biomarker Analysis

Biomarker data, as outlined in section 10.5.3, is being collected for all patients enrolled in this study. Blood biomarkers at baseline, day 2, day 6, and day 14/discharge/primary endpoint (+/- 2 days).

The objective will be to assess the SD of the biomarker data to enable power calculations to be performed and plan a future interim analysis based on biomarker values. The IDMC recommendations will include:

- whether a future biomarker interim analysis required
- timing of a future biomarker interim analysis
- which biomarkers to use
- scope and criteria for future decisions: early stopping for futility and/or efficacy

30 patients in each arm (who have completed 14 days/discharge/met the primary endpoint) is an adequate sample size to assess the SD of an endpoint. The data will be analysed within each treatment arm, but results will be anonymised and treatments given a random label "A", "B","C" when presented to the IDMC.

In the unlikely scenario that the sample size required for a future biomarker interim analysis is greater than 125 per active arm, then the futility analyses will be as described in section 15.2.3.

15.2.3 Clinical Efficacy Interim Analyses

An interim analysis will be scheduled after approximately 125 patients are recruited to each of the arms. A STOP/GO decision will be made by the IDMC based on the totality of data, including primary outcome, secondary clinical efficacy data, biomarkers, and safety data. This decision will be informed by Bayesian posterior distributions for the treatment effects on the primary outcome of each experimental treatment. Specifically, the IDMC will be provided with estimates of the probabilities for each treatment arm relative to control relating to efficacy **(HR≤1),** moderate or

greater efficacy (**HR<0.8**), similarity (**0.8 <HR< 1.25**) and harm (**HR≥1**). . The standard care arm will always be continued.

To inform the choice, Bayesian posterior distributions will be inferred for the treatment effects of each experimental treatment, assuming vague priors. These will be used to calculate, for each of the possible combination of arms to continue, predictive posterior probabilities that a future trial with a fixed total number of participants will result in any statistically significant positive results using a conventional 2.5% 1-sided hypothesis test: a Bayesian equivalent to predictive power. The total number of patients used for these calculations will be varied for consideration by the IDMC, in the light of recruitment rates, but a reference case will be a two armed trial comparing a treatment to standard of care, with a total N=229. The choice that provides the highest probability of ultimately achieving a significant result may be the recommendation, but the totality of information including external information will be considered by the IDMC. An arm may be stopped early for efficacy if Pocock's bounds are exceeded (p<0.001). An arm may be stopped early for futility, outside of the predictive posterior probability framework, if the posterior probability of a negative treatment effect exceeds 80%, or a clear safety signal is observed.

The IDMC will be presented with data relating to the primary outcome, the ordinal clinical status measure, and safety data. If the results are deemed inconclusive (e.g. there is a clear signal for efficacy but a potential signal for harm), the IDMC may recommend progressing. The subsequent timing of any future interim analyses will be determined by the IDMC.

15.3 Number of Participants to be enrolled

There is not a fixed sample size for this study and futility analyses will be performed to minimise the number of subjects exposed to potentially inefficacious compounds. As the agents in this study are either novel (pre-approval) or novel combinations of approved agents, a biomarker based futility analysis will be assessed to provide a very early stopping criteria. The point at which this early biomarker futility analysis can be performed will be determined during the study. If this futility analysis is successfully studied, then a second futility analysis will be performed after approximately 125 patients have been recruited to the active arm. Given the randomisation ratios, we would expect 375 patients to be recruited at the time of this analysis. Thereafter, the IDMC has the ability to recommend which arms are to continue or to allow additional arms, and for how many subsequent patients to recruit before the next interim analysis. Provisionally, the final analysis will be performed after 229 patients per active arm have been recruited. However the IDMC have the potential to advise recruiting up to a further 240 subjects to a maximum of 469 per active arm based on the observed data and as defined in the IDMC charter. Therefore, the expected number of subjects per arm is 229 and the maximum number of subjects per arm is 469.

The provisional target sample size of 229 per arm is based on a reference study with a fixed sample size involving two arms comparing treatment to standard of care, which is similar to other trials in this population. Specifically, 229 per arm is based on 80% power to detect a moderate clinically relevant difference in the primary outcome (one-sided alpha=.025) and also a key secondary outcome,

namely the ordinal clinical status at 14 days. The use Pocock's bounds for stopping for efficacy does not require adjustment for multiple testing.

i) The target sample size will allow for a detection of a 40% lower hazard (HR=.6) of the composite endpoint involving the first observed of invasive mechanical ventilation, renal replacement therapy, inotropic support, balloon pump, or death. This assumes the event rate in the control group is 20% at 14 days, which is consistent with the numbers requiring invasive mechanical ventilation or dying for the first 200 admission with COVID-19 at King's College Hospital. The expected event rate in the treatment group is approximately 12%.

ii) The target sample size will allow for a detection of 60% increased odds of better clinical status at 14 days on the 7-point COVID-19 ordinal outcomes scale. Based on data from the first 200 admission with COVID-19 at King's College Hospital, this represents an expected difference in the number scoring either of the two highest points on the scale, representing discharge, of 55.0% in the control group and 66.5% in the experimental group, and an expected difference in the number scoring the lowest point on the scale, death, of 15% in the control group and 9.8% in the experimental group.

The effects for each period before the interim analysis are given below, where we assume 125 patients per arm are recruited by 4 weeks, and the subsequent periods are four weeks each.

	Estimated Number of Patients per arm	Hazard Ratio, 80% power (Composite of events)	Odds ratio, 80% power (ordinal outcome scale)
Period 1	125	0.5	1.9
Period 2	229 (further 104)	0.6	1.6
Period 3	469 (further 240)	0.7	1.4

15.4 Procedure to account for missing or spurious data

Given data are collected mainly in hospital, with key outcome data being time to a fixed event (e.g. ICU admission or death), we anticipate the level of missing data to be negligible. Where missing data relates to an event, we will assume the event has not occurred up to the last known data point available for the patient, with the data censored at that point and not included in the further analyses. A similar approach will be undertaken for longitudinal mixed models under the assumption that data are missing at random. Only patients with at least one data point post-randomisation will be included in the analyses.

15.5 Definition of the end of the trial

The end of trial will be the date 18 months after the last patient's last visit to allow sufficient time to complete all primary, secondary, and exploratory endpoints and their corresponding analyses, and if applicable, all re-analyses of samples.

16 Data handling and record keeping

16.1 CRF

All data will be transferred into a Case Report Form (CRF) which will be labelled using a participant's unique trial ID and partial date of birth. All trial data in the CRF must be extracted from and be consistent with the relevant source documents. The CRFs must be completed, dated and signed by the investigator or designee in a timely manner. It remains the responsibility of the investigator for the timing, completeness, legibility and accuracy of the CRF pages. The CRF will be accessible to trial coordinators, data managers, the investigators, Clinical Trial Monitors, Auditors and Inspectors as required.

The investigator will retain the original of each completed CRF page at site. Completed CRFs should be emailed to the trial coordination centre. The investigator will also supply the trial coordination centre with any required, anonymised background information from the medical records as required.
A trial specific data management plan will describe in detail the data management processes using the CRF and the trial database.
Any trial related documentation that is sent to the trial coordination centre must not contain patient identifiable data.

All CRF pages must be clear, legible and completed in ink. Any errors should be crossed with a single stroke so that the original entry can still be seen. Corrections should be inserted and the change dated and initialled by the investigator or designee. If it is not clear why the change has been made, an explanation should be written next to the change. Typing correction fluid must not be used.

16.2 Source Data

To enable peer review, monitoring, audit and/or inspection the investigator must agree to keep records of all participating participants (sufficient information to link records e.g., CRFs, hospital records and samples), all original signed informed consent forms and copies of the CRF pages.

Source data may include but is not limited to:

- Signed informed consent forms
- Patient medical records (electronic or paper)
- Blood and imaging results (electronic or paper)
- Prescriptions
- Sample logs
- Clinical research forms/folders

16.3 Data Protection & Participant Confidentiality

All investigators and trial site staff involved in this trial must comply with the requirements of the General Data Protection Regulation and 2018 and Data Protection Act 2018 and Trust Policy with regards to the collection, storage, processing, transfer and disclosure of personal information and will uphold the Act's core principles.

17 Independent Data Monitoring Committee

The IDMC will be comprised of an independent group which meets regularly, as defined in a separate charter document which will define the role of the IDMC. The charter document will be generated prior to opening the trial.

The IDMC will be responsible for the review of all safety and efficacy as detailed below.

The IDMC will review the biomarker data after the first 10 subjects have reached Day 14/discharge/primary endpoint in each of the arms. This will be repeated after 30 per arm have reached Day 14/discharge/primary endpoint in each of the arms and again at 100 subjects per arm. The timings of subsequent analyses will be determined by the IDMC. In addition, the CI will call an *ad hoc* meeting at any point to review the data from an individual cohort if they are concerned about a potential safety signal.

Up to 3 futility analyses will be performed by the IDMC:

a. Biomarker based as defined by the initial biomarker assessment within the trial
b. Clinical based after 125 subjects in that arm have reached day 14
c. Clinical based after 229 subjects in that arm have reached day 14

If there is clear evidence of efficacy in any arm after 229 subjects have been treated then the IDMC may recommend that the data are published and the agent provided in the care pathway for COVID treatment. If clear evidence of efficacy is not observed but futility is also not observed after 229 subjects have reached day 14 then the IDMC may advise the recruitment of up to an additional 240 subjects.

A specified industry representative will be nominated from each industry partner to be a direct contact for the IDMC as a drug expert. These industry representatives will also be copied into any communication to the Ethics Committee/MHRA for any issues related to this study.

18 Trial Steering Committee

TSC will receive reports from the central co-ordination team at Cambridge CTU, TMG and IDMC. Full details of the TSC membership and remit can be found in the TSC Charter.

19 Ethical & Regulatory considerations

19.1 Ethical committee review

Before the start of the trial or implementation of any amendment we will obtain approval of the trial protocol, protocol amendments, informed consent forms and other relevant documents e.g., advertisements and GP information letters if applicable from the REC. All correspondence with the REC and HRA will be retained in the Trial Master File/Investigator Site File.

Annual reports will be submitted to the REC in accordance with national requirements. It is the Chief Investigator's responsibility to produce the annual reports as required.

19.2 Regulatory Compliance

The trial will not commence until a Clinical Trial Authorisation (CTA) is obtained from the MHRA. The protocol and trial conduct will comply with the Medicines for Human Use (Clinical Trials) Regulations 2004 and any relevant amendments.

Development Safety Update Reports (DSURs) will be submitted to the MHRA in accordance with national requirements. It is the Chief Investigators responsibility to produce the annual reports as required.

19.3 Protocol Amendments

Protocol amendments must be reviewed and agreement received from the Sponsor for all proposed amendments prior to submission to the HRA, REC and/or MHRA.

The only circumstance in which an amendment may be initiated prior to HRA, REC and/or MHRA approval is where the change is necessary to eliminate apparent, immediate risks to the participants (Urgent Safety Measures). In this case, accrual of new participants will be halted until the HRA, REC and/or MHRA approval has been obtained.

19.4 Peer Review

The TACTIC-E protocol has been reviewed by the TACTIC consortium as well as the Musculoskeletal and Respiratory Translational Research Collaboration (TRC) groups.

19.5 Declaration of Helsinki and Good Clinical Practice

The trial will be performed in accordance with the spirit and the letter of the declaration of Helsinki, the conditions and principles of Good Clinical Practice, the protocol and applicable local regulatory requirements and laws.

19.6 GCP Training

All trial staff must hold evidence of appropriate GCP training or undergo GCP training prior to undertaking any responsibilities on this trial. This training should be updated every 2 years or in accordance with your Trust's policy.

20 Sponsorship, Financial and Insurance

The trial is sponsored by Cambridge University Hospitals NHS Foundation Trust. The trial will be funded by AstraZeneca and Evelo Biosciences.

Cambridge University Hospitals NHS Foundation Trust, as a member of the NHS Clinical Negligence Scheme for Trusts, will accept full financial liability for harm caused to participants in the clinical trial caused through the negligence of its employees and honorary contract holders. There are no specific arrangements for

compensation should a participant be harmed through participation in the trial, but no-one has acted negligently.

21 Monitoring, Audit & Inspection

The investigator must make all trial documentation and related records available should an MHRA Inspection occur. Should a monitoring visit or audit be requested, the investigator must make the trial documentation and source data available to the Sponsor's representative. All participant data must be handled and treated confidentially.

The Sponsor's monitoring frequency will be determined by an initial risk assessment performed prior to the start of the trial. A detailed monitoring plan will be generated detailing the frequency and scope of the monitoring for the trial. Throughout the course of the trial, the risk assessment will be reviewed and the monitoring frequency adjusted as necessary.

Remote monitoring will be conducted for all participating sites. The scope and frequency of the monitoring will be determined by the risk assessment and detailed in the Monitoring Plan for the trial.

22 Protocol Compliance and Breaches of GCP

Prospective, planned deviations or waivers to the protocol are not allowed under the UK regulations on Clinical Trials and must not be used.

Protocol deviations, non-compliances, or breaches are departures from the approved protocol. They can happen at any time but are not planned. They must be adequately documented on the relevant forms and reported to the Chief Investigator and Sponsor immediately.

Deviations from the protocol which are found to occur repeatedly will not be accepted and will require immediate action and could potentially be classified as a serious breach.

Any potential/suspected serious breaches of GCP must be reported immediately to the Sponsor without any delay.

23 Publications policy

Ownership of the data arising from this trial resides with the trial team and the sponsor. On completion of the trial, the data will be analysed and tabulated and a Final Trial Report prepared. However, given the nature of this international pandemic, preliminary data may be reported prior to the completion of long term follow up of patients in the study, or if interim analyses are adequate for dissemination of critical safety or efficacy data. At conclusion of the study a fully anonymised dataset will be placed in the public domain. Data sharing within a federated consortium of UK investigators across the four nations will be adopted.

24 References

- Al-Omari et al 2018 MERS coronavirus outbreak: Implications for emerging viral infections. Diagnostic Microbiol and Infect Disease 93: 265-285; doi.org/10.1016/j.diagmicrobio.2018.10.011Guan et al 2020. Clinical Characteristics of Coronavirus Diseae 2019 in China. NEJM DOI: 10.1056/NEJMoa2002032
- Aragón-Herrera A, Feijóo-Bandín S, Otero Santiago M, Barral L, Campos-Toimil M, Gil-Longo J, et al. Empagliflozin reduces the levels of CD36 and cardiotoxic lipids while improving autophagy in the hearts of Zucker diabetic fatty rats. Biochem Pharmacol. 2019 Dec;170:113677.
- Arentz M, Yim E, Klaff L, Lokhandwala S, Riedo FX, Chong M, et al. Characteristics and Outcomes of 21 Critically Ill Patients With COVID-19 in Washington State. JAMA. 2020 Apr 28;323(16):1612–4.
- Chowdhury B, Luu VZ, Luu AZ, Kabir MG, Pan Y, Teoh H, et al. 56The SGLT2 inhibitor empagliflozin reduces mortality in experimental pulmonary hypertension. Eur Heart J [Internet]. 2019 Oct 1 [cited 2020 Apr 29];40(Supplement_1).
- Druml W, Steltzer H, Waldhäusl W, Lenz K, Hammerle A, Vierhapper H, et al. Endothelin-1 in adult respiratory distress syndrome. Am Rev Respir Dis. 1993 Nov;148(5):1169–73.
- Esterline RL, Vaag A, Oscarsson J, Vora J. MECHANISMS IN ENDOCRINOLOGY: SGLT2 inhibitors: clinical benefits by restoration of normal diurnal metabolism? Eur J Endocrinol. 2018 Apr;178(4):R113–25.
- Ferrannini E, Solini A. Therapy: SGLT inhibition in T1DM - definite benefit with manageable risk. Nat Rev Endocrinol. 2017;13(12):698–9.
- Gattinoni L, Chiumello D, Caironi P, Busana M, Romitti F, Brazzi L, et al. COVID-19 pneumonia: different respiratory treatments for different phenotypes? Intensive Care Med [Internet]. 2020 Apr 14 [cited 2020 Apr 29]; Available from: https://doi.org/10.1007/s00134-020-06033-2
- Gautret et al 2020. Clinical and microbiological effect of a combination of hydroxychloroquine and azithromycin in 80 COVID-19 patients with at least a six-day follow up: an observational study – published ahead of press by Lover, A. A. Quantifying treatment effects of hydroxychloroquine and azithromycin for COVID-19: a secondary analysis of an open label non-randomized clinical trial. medRxiv 2020.03.22.20040949 (2020) doi:10.1101/2020.03.22.20040949.
- Grasselli G, Pesenti A, Cecconi M. Critical Care Utilization for the COVID-19 Outbreak in Lombardy, Italy: Early Experience and Forecast During an Emergency Response. JAMA [Internet]. 2020 Mar 13 [cited 2020 Mar 27]; Available from: https://jamanetwork.com/journals/jama/fullarticle/2763188
- Gralinski and Baric 2015. Molecular pathology of emerging coronavirus infections. J Pathol 2015: 235: 185-195; DOI: 10.1002/path.4454
- Guo Q, Huang J-A, Fraidenburg DR. Bosentan as rescue treatment in refractory hypoxemia and pulmonary hypertension in a patient with ARDS and H7N9 influenza virus infection. Lung. 2014 Oct;192(5):635–6.
- Hele DJ, Birrell MA, Webber SE, Foster ML, Belvisi MG. Effect of endothelin antagonists, including the novel ET(A) receptor antagonist LBL 031, on

endothelin-1 and lipopolysaccharide-induced microvascular leakage in rat airways. Br J Pharmacol. 2000 Nov;131(6):1129–34.

- Huang C, Wang Y, Li X, Ren L, Zhao J, Hu Y, et al. Clinical features of patients infected with 2019 novel coronavirus in Wuhan, China. Lancet. 2020;395(10223):497–506.
- Kuklin V, Kirov M, Sovershaev M, Andreasen T, Ingebretsen OC, Ytrehus K, et al. Tezosentan-induced attenuation of lung injury in endotoxemic sheep is associated with reduced activation of protein kinase C. Crit Care Lond Engl. 2005 Jun;9(3):R211-217.
- McMurray JJV, DeMets DL, Inzucchi SE, Køber L, Kosiborod MN, Langkilde AM, et al. A trial to evaluate the effect of the sodium-glucose co-transporter 2 inhibitor dapagliflozin on morbidity and mortality in patients with heart failure and reduced left ventricular ejection fraction (DAPA-HF). Eur J Heart Fail. 2019 May;21(5):665–75.
- Neal B, Perkovic V, Mahaffey KW, Fulcher G, Erondu N, Desai M, et al. Optimizing the analysis strategy for the CANVAS Program: A prespecified plan for the integrated analyses of the CANVAS and CANVAS-R trials. Diabetes Obes Metab. 2017;19(7):926–35.
- Ohara, K., Masuda, T., Morinari, M. *et al.* The extracellular volume status predicts body fluid response to SGLT2 inhibitor dapagliflozin in diabetic kidney disease. *Diabetol Metab Syndr* **12,** 37 (2020). https://doi.org/10.1186/s13098-020-00545-z
- Packer M. Critical Examination of Mechanisms Underlying the Reduction in Heart Failure Events With SGLT2 Inhibitors: Identification of a Molecular Link Between Their Actions to Stimulate Erythrocytosis and to Alleviate Cellular Stress. Cardiovasc Res. 2020 Apr 3;
- Peiris et al 2003. Clinical progression and viral load in a community outbreak of coronavirus-associated SARS pneumonia: a prospective study. Lancet 361: 1767-72
- Perkovic V, Jardine MJ, Neal B, Bompoint S, Heerspink HJL, Charytan DM, et al. Canagliflozin and Renal Outcomes in Type 2 Diabetes and Nephropathy. N Engl J Med. 2019 13;380(24):2295–306.
- Russell, C. D., Millar, J. E. & Baillie, J. K. Clinical evidence does not support corticosteroid treatment for 2019-nCoV lung injury. The Lancet 395, 473–475 (2020).
- Shinagawa S, Okazaki T, Ikeda M, Yudoh K, Kisanuki YY, Yanagisawa M, et al. T cells upon activation promote endothelin 1 production in monocytes via IFN-γ and TNF-α. Sci Rep. 2017 Nov 3;7(1):1–8.
- Tanaka S, Sugiura Y, Saito H, Sugahara M, Higashijima Y, Yamaguchi J, et al. Sodium-glucose cotransporter 2 inhibition normalizes glucose metabolism and suppresses oxidative stress in the kidneys of diabetic mice. Kidney Int. 2018;94(5):912–25.
- Zelniker TA, Wiviott SD, Raz I, Im K, Goodrich EL, Bonaca MP, et al. SGLT2 inhibitors for primary and secondary prevention of cardiovascular and renal outcomes in type 2 diabetes: a systematic review and meta-analysis of cardiovascular outcome trials. Lancet Lond Engl. 2019 05;393(10166):31–9.

- Zhou et al 2020 Utility of Ferritin, Procalcitonin and C-reactive protein in Severe Patients with 2019 Novel Coronavirus Disease. 10.21203/rs.3.rs-18079/v1

Safety Reporting Flow Chart

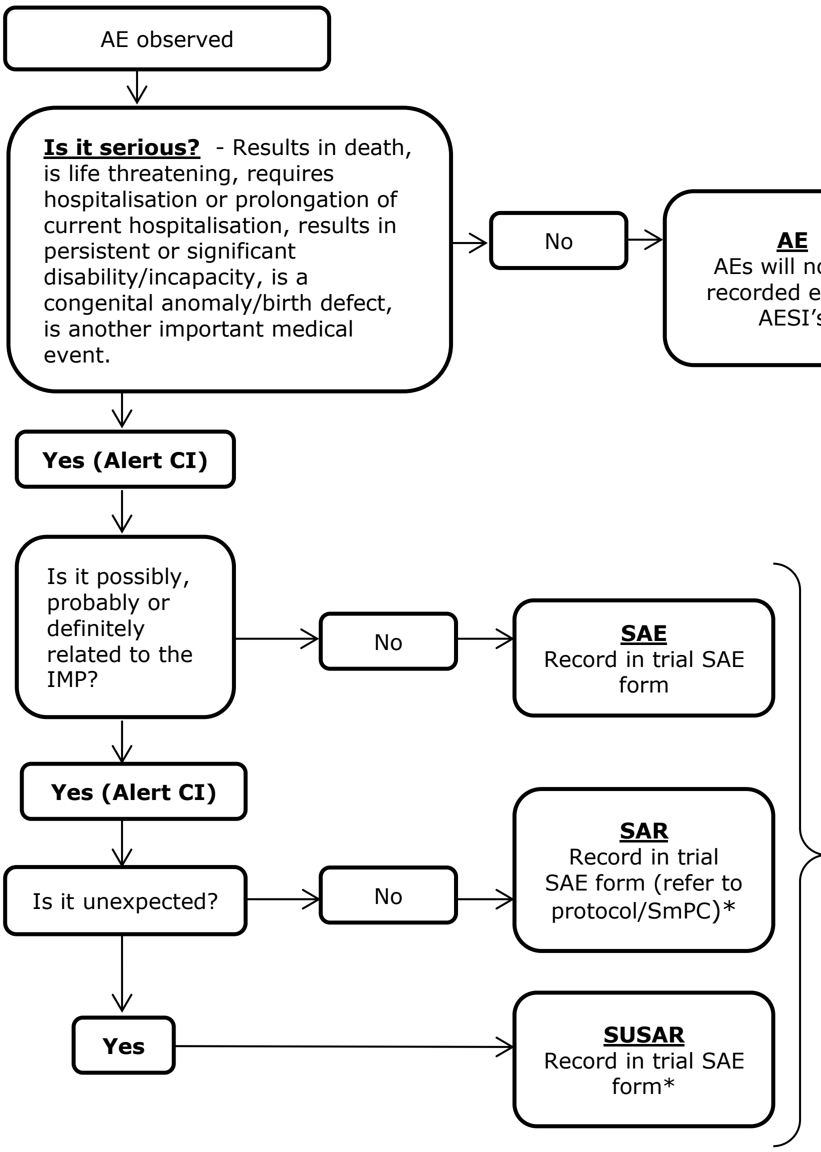

SAE/SAR/SUSAR reporting
<u>Initial report</u><br>Within <u>24 hours</u> of investigator awareness report to:<br><u>Coordinator:</u><br>Cambs.cardiovascular@nhs.net,<br>cctupharmacovigilance@addenbrookes.nhs.uk,<br>SAE/SAR/SUSARs for the Dapglifozin/Ambrisentan arm should ALSO be sent to:<br>AEMailboxClinicalTrialTCS@astrazeneca.com<br>SAE/SAR/SUSARs for EDP1815 should ALSO be sent to medpace-safetynotification@medpace.com.

SUSAR expedited reporting
The Chief Investigator must report a SUSAR to the Sponsor, REC and MHRA within statutory timelines. Each SUSAR requires the entry of relevant data and information by the Chief Investigator into the eSUSAR reporting system. A copy of this report should be provided to the CCTU PV team, collating these on behalf of the sponsor.

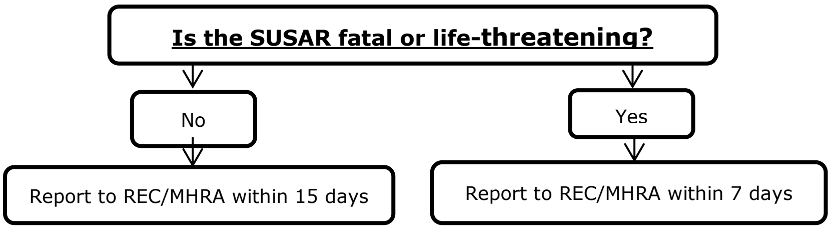

Supplement: Supplementary file 1 — Additional file 1. Full Study Protocol. [file 13063_2020_4618_MOESM1_ESM.pdf]
